# Discovery and Validation of Circulating microRNAs as Biomarkers for Epileptogenesis after Experimental Traumatic Brain Injury–The EPITARGET Cohort

**DOI:** 10.3390/ijms24032823

**Published:** 2023-02-01

**Authors:** Mette Heiskanen, Shalini Das Gupta, James D. Mills, Erwin A. van Vliet, Eppu Manninen, Robert Ciszek, Pedro Andrade, Noora Puhakka, Eleonora Aronica, Asla Pitkänen

**Affiliations:** 1 A.I. Virtanen Institute for Molecular Sciences, University of Eastern Finland, 70211 Kuopio, Finland; 2 Department of (Neuro)Pathology, Amsterdam UMC, University of Amsterdam, 1105 AZ Amsterdam, The Netherlands; 3 Department of Clinical and Experimental Epilepsy, UCL Queen Square Institute of Neurology, London WC1N 3BG, UK; 4 Chalfont Centre for Epilepsy, Buckinghamshire SL9 0RJ, UK; 5 Swammerdam Institute for Life Sciences, Center for Neuroscience, University of Amsterdam, 1098 XH Amsterdam, The Netherlands; 6 Stichting Epilepsie Instellingen Nederland, 2103 SW Heemstede, The Netherlands

**Keywords:** fluid-percussion injury, post-traumatic epilepsy, rat, plasma

## Abstract

Traumatic brain injury (TBI) causes 10–20% of structural epilepsies and 5% of all epilepsies. The lack of prognostic biomarkers for post-traumatic epilepsy (PTE) is a major obstacle to the development of anti-epileptogenic treatments. Previous studies revealed TBI-induced alterations in blood microRNA (miRNA) levels, and patients with epilepsy exhibit dysregulation of blood miRNAs. We hypothesized that acutely altered plasma miRNAs could serve as prognostic biomarkers for brain damage severity and the development of PTE. To investigate this, epileptogenesis was induced in adult male Sprague Dawley rats by lateral fluid-percussion-induced TBI. Epilepsy was defined as the occurrence of at least one unprovoked seizure during continuous 1-month video-electroencephalography monitoring in the sixth post-TBI month. Cortical pathology was analyzed by magnetic resonance imaging on day 2 (D2), D7, and D21, and by histology 6 months post-TBI. Small RNA sequencing was performed from tail-vein plasma samples on D2 and D9 after TBI (*n* = 16, 7 with and 9 without epilepsy) or sham operation (*n* = 4). The most promising miRNA biomarker candidates were validated by droplet digital polymerase chain reaction in a validation cohort of 115 rats (8 naïve, 17 sham, and 90 TBI rats [21 with epilepsy]). These included 7 brain-enriched plasma miRNAs (miR-434-3p, miR-9a-3p, miR-136-3p, miR-323-3p, miR-124-3p, miR-212-3p, and miR-132-3p) that were upregulated on D2 post-TBI (*p* < 0.001 for all compared with naïve rats). The acute post-TBI plasma miRNA profile did not predict the subsequent development of PTE or PTE severity. Plasma miRNA levels, however, predicted the cortical pathology severity on D2 (Spearman *ρ* = 0.345–0.582, *p* < 0.001), D9 (*ρ* = 0.287–0.522, *p* < 0.001–0.01), D21 (*ρ* = 0.269–0.581, *p* < 0.001–0.05) and at 6 months post-TBI (*ρ* = 0.230–0.433, *p* < 0.001–0.05). We found that the levels of 6 of 7 miRNAs also reflected mild brain injury caused by the craniotomy during sham operation (ROC AUC 0.76–0.96, *p* < 0.001–0.05). In conclusion, our findings revealed that increased levels of neuronally enriched miRNAs in the blood circulation after TBI reflect the extent of cortical injury in the brain but do not predict PTE development.

## 1. Introduction

Worldwide, it is estimated that >50 million people have epilepsy and 5 million people are diagnosed with epilepsy each year, meaning that a new epilepsy diagnosis is made every 6 s (WHO, https://www.who.int/publications/i/item/who-information-kit-on-epilepsy, accessed on 14 November 2022). Traumatic brain injury (TBI) causes 10–20% of structural epilepsies and 5% of all epilepsies [1]. TBI is defined as an alteration in brain function or other brain pathology caused by an external force [2]. The risk of post-traumatic epilepsy (PTE) increases with TBI severity: the 30-year cumulative incidence rate for PTE is 2% for mild, 4% for moderate, and 17% for severe TBI [3]. The latency period from the initial injury to the appearance of the first seizures can vary from months to years [3]. Currently, there are no available treatments for stopping or preventing the epileptogenic process [4,5]. The major obstacle in developing anti-epileptogenic treatments is the lack of prognostic biomarkers, hindering the stratification of subjects with the highest risk into preclinical and clinical studies. Therefore, testing of candidate anti-epileptogenic treatments remains laborious and expensive [6]. Identifying prognostic biomarkers for the development of PTE after TBI is a major unmet medical need.

The major modalities investigated to date as a source of prognostic biomarkers for PTE include brain imaging, electroencephalography (EEG), and blood or cerebrospinal fluid (CSF) [5]. Blood sampling is minimally invasive, making circulating molecules such as microRNAs (miRNAs) an appealing source of disease biomarkers. MiRNAs are short (~22 nucleotides) non-coding RNAs that regulate gene expression at the post-transcriptional level by binding to the 3′ untranslated region of their target mRNAs [7,8]. MiRNAs are estimated to regulate more than 60% of all human proteins [9], and many miRNAs are specifically expressed in the brain [10,11,12]. Thus, circulating brain-enriched miRNAs provide an attractive source, not only for diagnosis but also for longitudinally monitoring brain pathology progression.

Accumulating evidence indicates dysregulation of miRNAs in the brain tissue of patients with epilepsy [13,14]. The dysregulated miRNAs are proposed to be involved in many epileptogenesis-related brain pathologies, such as neuroinflammation and synaptic plasticity [14]. Consistent with miRNA dysregulation in the brain, circulating miRNAs are altered in patients with epilepsy [15]. These data provide a promising scenario that alterations in brain-enriched miRNAs in the blood circulation after TBI could reflect ongoing epileptogenic processes before the onset of PTE. Importantly, preclinical and clinical studies revealed TBI-induced alterations in circulating miRNAs [15,16]. It remains to be explored, however, whether the post-TBI alterations in circulating miRNA profiles provide information on the risk of developing PTE.

We hypothesized that plasma miRNAs at the acute post-TBI time-point will present prognostic biomarkers for brain damage severity and the development of PTE. The data presented derive from a uniquely large and well-characterized animal cohort (EPITARGET, [17]) in which TBI was induced by lateral fluid-percussion injury (LFPI) and epilepsy phenotyping was conducted by 1-month video-electroencephalography (vEEG) monitoring during the sixth month after TBI. The three main objectives were to: (a) discover differentially expressed plasma miRNA profiles after TBI; (b) investigate associations between plasma miRNA levels and the cortical damage severity; and (c) determine whether the plasma miRNA profile at an acute post-TBI time-point could be used as a biomarker to predict epileptogenesis or epilepsy severity after TBI.

## 2. Results

### 2.1. MiRNA Sequencing

#### 2.1.1. MiRNA Quantification

**Figure 1:**
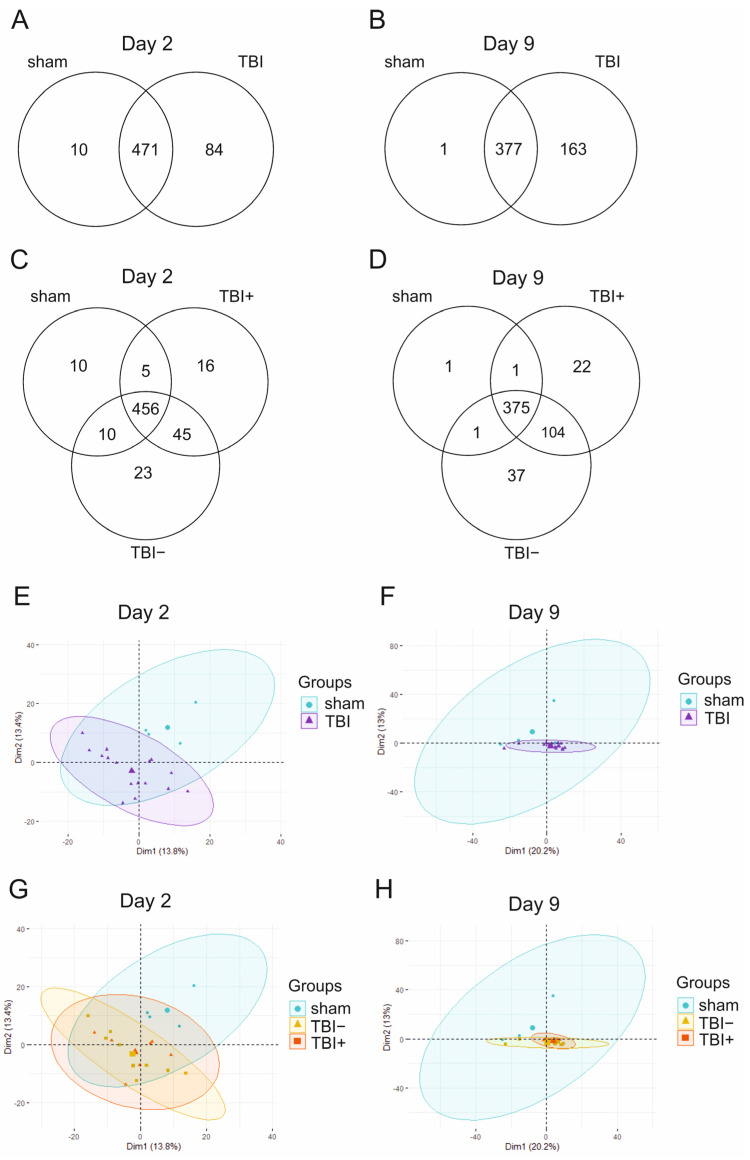
Venn diagrams and principal component analysis (PCA) plots of miRNAs detected in rat plasma by small RNA sequencing. (**A**) A total of 565 miRNAs were detected in rat plasma on day 2 (D2), 471 of which were common between sham and TBI rats. There were 84 miRNAs detected only in the TBI group. (**B**) A total of 541 miRNAs were detected in rat plasma on D9, 377 of which were common between sham and TBI rats. There were 163 miRNAs detected only in the TBI group. (**C**) On D2, 16 miRNAs were detected in the TBI animals with epilepsy (TBI+) but not in the sham group or in TBI animals without epilepsy (TBI−). These miRNAs, however, were typically found in only 1 sample in the TBI+ group, and the number of reads was very low (<3). (**D**) On D9, 22 miRNAs were detected in the TBI+ group but not in the sham and TBI− groups. As on D2, these miRNAs were typically found in only 1 sample in the TBI+ group and had a very low read number. (**E**–**H**) According to PCA, the first and second principal components explained 27% of the variance in the data on D2 and 33% on D9. Plasma miRNA expression profile separated sham and TBI rats into different clusters on D2 but not on D9. The miRNA expression profile did not separate TBI+ and TBI− rats at either time-point.

***Read counts.*** MicroRNA sequencing was performed on D2 and D9 plasma samples of the discovery cohort, including 4 sham-operated controls and 16 rats with TBI (7 with epilepsy [TBI+], 9 without epilepsy [TBI−]). MicroRNA sequencing detected a total of 565 miRNAs in at least one D2 sample and 541 miRNAs in at least one D9 sample (Figure 1A,B). In D2 samples, the number of mapped reads did not differ between the groups. In the D9 samples, the number of mapped reads in the sham-operated group was only 17% of that in the TBI animals (Mann–Whitney U test, *p* < 0.001) (Supplementary Figure S1).

***Principal component analysis (PCA).*** On D2, the PCA of normalized read counts (counts/million) revealed that the first (14%) and second (13%) principal components explained only 27% of the variance in the data (Figure 1E,G). The plasma miRNA expression profile on D2 separated sham and TBI rats into different clusters (Figure 1E). On D9, the first (20%) and second (13%) components explained 33% of the data variance (Figure 1F,H). The plasma miRNA expression profile on D9 did not separate sham and TBI rats (Figure 1F). The miRNA expression profile did not separate TBI+ and TBI− at either time-point (Figure 1G,H).

***Spearman correlation analysis*** yielded a high positive correlation between miRNA expression profiles across all samples on both D2 and D9 (Supplementary Figure S2).

***Heatmaps.*** On D2, heatmap analysis of miRNA expression profiles in all samples showed a separation between sham and TBI animals (Supplementary Figure S3). Pairwise comparisons revealed a clear separation between sham and TBI, sham and TBI+, and sham and TBI− groups (Supplementary Figure S4). No separation, however, was detected between the TBI+ and TBI− animals.

On D9, heatmap analysis of miRNA expression profiles in all samples revealed no clear separation between sham and TBI samples (Supplementary Figure S3). In addition, pairwise comparisons revealed no clear separation between groups (Supplementary Figure S5). As on D2, no separation was detected between the TBI+ and TBI− animals on D9.

#### 2.1.2. Differential Expression Analysis

Differentially expressed miRNAs are presented in the Supplementary Materials (Tables S1–S6).

***D2 samples.*** DESeq2 analysis detected 45 differentially expressed miRNAs between the TBI and sham groups (28 upregulated, 17 downregulated), 34 between the TBI+ and sham groups (27 upregulated, 7 downregulated), and 37 between the TBI− and sham groups (21 upregulated, 16 downregulated). No differentially expressed miRNAs were detected between the TBI+ and TBI− groups on D2.

***D9 samples.*** DESeq2 analysis detected 17 differentially expressed miRNAs between the TBI and sham groups, (6 upregulated, 11 downregulated), 11 between the TBI+ and sham groups (2 upregulated, 9 downregulated), and 22 between the TBI− and sham groups (8 upregulated, 14 downregulated). No differentially expressed miRNAs were detected between the TBI+ and TBI− groups on D9.

#### 2.1.3. Expression Pattern Differences from Machine Learning Analysis

The feature importance from logistic regression analysis for the miRNA candidates differentiating the groups is presented in Supplementary Figure S6.

***D2 samples.*** On D2, logistic regression analysis differentiated between TBI and sham groups with the cross-validated area under the curve (CV AUC) 0.94. Among these miRNAs, 23/30 were also identified by differential expression analysis. Logistic regression analysis did not differentiate TBI+ and TBI− on D2 (CV AUC 0.50).

***D9 samples.*** On D9, logistic regression analysis differentiated between TBI and sham groups with a cross-validated AUC 0.94. None of the 29 miRNAs from the logistic regression analysis were identified by the differential expression analysis. Logistic regression analysis did not differentiate TBI+ and TBI− on D9 (CV AUC 0.31).

### 2.2. Technical Validation with RT-qPCR–Discovery Cohort

As the heatmap analysis of miRNA expression profiles from miRNA sequencing indicated poor separation of the TBI and sham groups on D9, the subsequent technical validation (TBI vs. sham) focused on D2 sequencing data in samples available from the discovery cohort.

#### 2.2.1. Selection of Differentially Expressed miRNAs for PCR Validation in D2 Samples

Of the 28 upregulated miRNAs (TBI vs. sham) in the miRNA-sequencing analysis, we selected 3 miRNAs (miR-136-3p, miR-323-3p, and miR-129-5p) for technical validation by RT-qPCR. The selection was based on log2FC ≥1.0, a low adjusted p-value, and a mean CPM ≥ 30. In addition, we validated two other miRNAs (miR-434-3p and miR-9a-3p), which we earlier identified as strongly upregulated on D2 after TBI [18], to assess the consistency of findings between the studies and their potential as biomarkers for epileptogenesis.

#### 2.2.2. Quantitative Reverse Transcription PCR

**Figure 2:**
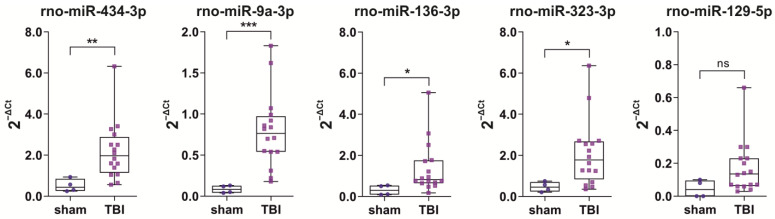
Technical validation of 5 plasma miRNA levels by RT-qPCR. Box and whisker plots (whiskers: minimum and maximum; box: interquartile range; line: median) showing the levels of 5 miRNAs (miR-434-3p, miR-9a-3p, miR-136-3p, miR-323-3p, and miR-129-5p) in rat plasma on D2 (48 h) after TBI or sham-operation. All of the miRNAs except miR-129-5p were upregulated in the TBI group *(n* = 16) compared with the sham group (*n* = 4). Expression levels were normalized to the endogenous control miR-28-3p with the 2^−ΔCt^ method. Statistical significance: *, *p* < 0.05; **, *p* < 0.01; ***, *p* < 0.001 (Mann–Whitney U test). Abbreviations: ns, not significant; TBI, traumatic brain injury.

***TBI* vs. *sham.*** Quantitative reverse transcription PCR (RT-qPCR) results are summarized in Figure 2. Technical validation of the 3 miRNAs (miR-139-3p, miR-323-3p, miR-129-5p) included different aliquots of plasma samples collected from the same 16 TBI rats and 4 sham-operated controls that were used for small RNA sequencing. Although miR-434-3p and miR-9a-3p were not detected in small RNA sequencing, both were detected by RT-qPCR. **miR-434-3p**. The miR-434-3p levels were 4.2-fold higher in the TBI group than in the sham group (2.20 ± 1.41 vs. 0.52 ± 0.31, *p* < 0.01). **miR-9a-3p.** The miR-9a-3p levels were 9.3-fold higher in the TBI group than in the sham group (0.79 ± 0.45 vs. 0.09 ± 0.05, *p* < 0.001). **miR-136-3p**. The miR-136-3p levels were 4.4-fold higher in the TBI group than in the sham group (1.37 ± 1.26 vs. 0.31 ± 0.24, *p* < 0.05). **miR-323-3p**. The miR-323-3p levels were 4.5-fold higher in the TBI group than in the sham group (2.08 ± 1.61 vs. 0.47 ± 0.24, *p* < 0.05). **miR-129-5p**. The miR-129-5p levels were 1.9-fold higher in the TBI group than in the sham group (0.17 ± 0.16 vs. 0.05 ± 0.05), but the difference was not statistically significant (*p* > 0.05). Because the miR-129-5-p expression levels were very low and did not differ between the sham and TBI groups, miR-129-5p was excluded from the subsequent ddPCR-based analysis in the validation cohort. 

***TBI+ vs*. *TBI−.*** No differences were detected in any of the five miRNAs assessed with RT-qPCR (*p* > 0.05) between the TBI+ (*n* = 7) and TBI− (*n* = 9) groups.

### 2.3. D2 Plasma Levels of miRNAs in the Validation Cohort

#### 2.3.1. Selection of miRNAs for ddPCR Analysis

***Upregulated miRNAs.*** Based on the miRNA sequencing, we made a list of brain-specific or brain-enriched miRNAs with log2FC ≥ 1.0 and a low *p*-value [PubMed search, Human miRNA tissue atlas [12]; RATEmiRs database [19]. Preliminary experiments revealed that miRNAs on that list with a CPM < 30 were difficult to validate with ddPCR. Therefore, we focused on miRNAs with a CPM ≥ 30 in the TBI group. Consequently, miR-434-3p, miR-9a-3p, miR-136-3p, and miR-323-3p were included in the final analysis based on the miRNA-sequencing and technical validation. The list was complemented by three additional miRNAs, including miR-124-3p (log2FC 7.4, CPM 31), miR-212-3p (log2FC 1.3, CPM 39), and miR-132-3p (log2FC 1.4, CPM 67), which we anticipated being able to quantify by ddPCR.

***Downregulated miRNAs.*** We also performed preliminary experiments using ddPCR of three miRNAs that were downregulated in miRNA sequencing and showed the lowest adjusted *p*-value (miR-455-5p [log2FC −1.5, CPM 107 in the TBI group], miR-140-3p [log2FC −1.1, CPM 5930 in the TBI group], and miR-149-5p [log2FC −1.3, CPM 248 in the TBI group]) (Supplementary Table S1). **miR-455-5p**. On D2, normalized plasma levels of miR-455-5p in the TBI group were only 40% of that in naïve rats (0.92 ± 0.21 vs. 2.32 ± 0.88, *p* < 0.01) (Supplementary Figure S7A). In addition, miR-455-5p levels showed a decreasing trend in the sham group compared with naïve animals (1.04 ± 0.48 vs. 2.32 ± 0.88, 55% decrease; *p* > 0.05). No difference was detected, however, between the TBI and sham groups (11% decrease, *p* > 0.05). **miR-140-3p** or **miR-149-5p** levels did not differ between the experimental groups (Kruskal–Wallis test, *p* > 0.05), although there was a decreasing trend in the order of naïve>sham or TBI (Supplementary Figure S7B,C).

Based on these data, we focused our search of plasma miRNA biomarkers on the list of the seven upregulated miRNAs.

#### 2.3.2. Sample Quality

***Plasma quality–hemolysis measurement by NanoDrop.*** Altogether, 115 of the 150 plasma samples (77%) available on D2 from the epilepsy-phenotyped rats qualified for ddPCR analysis (sufficient volume of plasma and A414 < 0.25). The mean A414 in the 115 pooled plasma samples was 0.13 ± 0.04 (range 0.05–0.24, median 0.14) (Supplementary Figure S8A). Animals with TBI (*n* = 90) had a slightly lower A414 than naïve animals (*n* = 8; 0.130 vs. 0.162, *p* < 0.05) (Supplementary Figure S8B).

***Small RNA concentration by Qubit assay.*** In plasma samples collected from the validation cohort, the mean small RNA concentration after RNA extraction was 419 ng/mL ± 288 ng/mL (*n* = 111; range: 4.9 ng/mL–1471 ng/mL, median: 343 ng/mL. Four samples (two naïve, one TBI−, one TBI+) had miRNA concentrations below the Qubit detection limit, but they could still be analyzed by ddPCR and were included in the analysis. We detected no differences in small RNA concentration between naïve, sham, and TBI groups (Kruskal–Wallis test, *p* > 0.05) (Supplementary Figure S6C).

***Correlation between hemolysis and small RNA concentration.*** A weak positive correlation was detected between the A414 values and small RNA concentration measured by Qubit (*n* = 111, Spearman r = 0.327, *p* < 0.001), that is, the higher the A414 value, the greater the small RNA concentration (Supplementary Figure S8D). We detected no correlation, however, between the plasma A414 value and unnormalized miRNA expression levels of any of the analyzed miRNAs (*n* = 115, Spearman correlation, *p* > 0.05 for all), indicating that hemolysis did not affect miRNA expression levels.

### 2.4. D2 Plasma miRNA Levels in Different Treatment Groups

#### 2.4.1. Naïve vs. Sham vs. TBI

**Figure 3:**
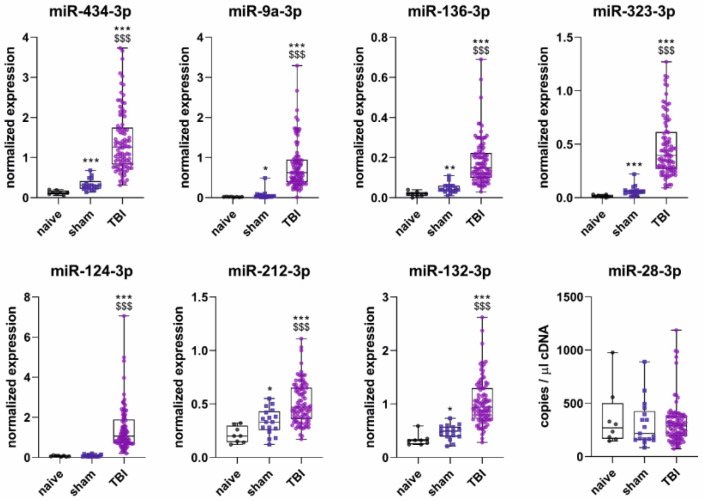
D2 plasma miRNA levels in different animal groups. Box and whisker plots (whiskers: minimum and maximum; box: interquartile range; line: median) showing the normalized expression levels of 7 validated miRNAs (miR-434-3p, miR-9a-3p, miR-136-3p, miR-323-3p, miR-124-3p, miR-212-3p, and miR-132-3p) measured by droplet digital PCR (ddPCR). MiR-28-3p was used as endogenous reference for normalization (target concentration/reference concentration). All 7 miRNAs were upregulated in the TBI group (*n* = 90) compared with naïve (*n* = 8) or sham-operated (*n* = 17) rats. Of 7 miRNAs, 6 (all except miR-124-3p) were upregulated in the sham group compared with the naïve group. The levels of the endogenous control miR-28-3p did not differ between the animal groups. Statistical significance: *, *p* < 0.05; **, *p* < 0.01; ***, *p* < 0.001 (compared with naïve); $$$, *p* < 0.001 (compared with sham; Mann–Whitney U test). Abbreviations: TBI, traumatic brain injury.

The plasma concentrations of the seven miRNAs in the validation cohort are summarized in Figure 3. The validation cohort included 8 naïve, 17 sham, and 90 TBI rats (21 TBI+, 69 TBI−). As the samples had variable small RNA concentrations after RNA extraction, the target miRNA expression levels in each sample were normalized to the endogenous control miR-28-3p (target miRNA copy number/miR-28-3p copy number). The mean copy number of miR-28-3p was similar between the different experimental groups (Kruskal–Wallis test, *p* > 0.05). 

**miR-434-3p.** The average normalized plasma levels of miR-434-3p in the sham-operated controls were 2.3-fold higher than in naïve rats (0.33 ± 0.15 vs. 0.13 ± 0.05, *p* < 0.001). TBI rats had 11.2-fold higher normalized miR-434-3p levels than naïve rats (1.41 ± 0.77 vs. 0.13, *p* < 0.001) and 4.3-fold higher levels than sham-operated controls (1.41 vs. 0.33, *p* < 0.001). 

**miR-9a-3p**. Sham-operated controls had 3.4-fold higher normalized levels of plasma miR-9a-3p than naïve rats (0.07 ± 0.11 vs. 0.02 ± 0.008, *p* < 0.05). TBI rats had 41.1-fold higher miR-9a-3p levels than naïve rats (0.81 ± 0.59 vs. 0.02, *p* < 0.001) and 22.4-fold higher levels than sham-operated controls (0.81 vs. 0.07, *p* < 0.001).

**miR-136-3p**. The sham group had 2.9-fold higher normalized levels of plasma miR-136-3p than the naïve group (0.05 ± 0.02 vs. 0.02 ± 0.01, *p* < 0.01). TBI rats had 10.6-fold higher miR-136-3p levels than naïve rats (0.18 ± 0.11 vs. 0.02, *p* < 0.001) and 3.7-fold higher levels than sham-operated controls (0.18 vs. 0.05, *p* < 0.001). 

**miR-323-3p**. Sham-operated controls had 4.2-fold higher normalized levels of plasma miR-323-3p levels than naïve rats (0.06 ± 0.05 vs. 0.02 ± 0.01, *p* < 0.001). TBI rats had 30.6-fold higher miR-323-3p levels than naïve rats (0.47 ± 0.28 vs. 0.02, *p* < 0.001) and 7.3-fold higher levels than sham-operated controls (0.47 vs. 0.06, *p* < 0.001). 

**miR-124-3p**. Sham-operated controls and naïve rats had similar normalized levels of plasma miR-124-3p (0.08 ± 0.05 vs. 0.07 ± 0.03, *p* > 0.05). TBI rats had 20.8-fold higher miR-124-3p levels than naïve rats (1.41 ± 1.11 vs. 0.07, *p* < 0.001) and 18.2-fold higher levels than sham-operated controls (1.41 vs. 0.08, *p* < 0.001). 

**miR-212-3p**. Sham-operated controls had 1.6-fold higher normalized levels of plasma miR-212-3p than naïve rats (0.34 ± 0.12 vs. 0.21 ± 0.08, *p* < 0.05). TBI rats had 2.4-fold higher miR-212-3p levels than naïve rats (0.51 ± 0.19 vs. 0.21, *p* < 0.001 and 1.5-fold higher levels than sham-operated controls (0.51 vs. 0.34, *p* < 0.001). 

**miR-132-3p**. Sham-operated controls had 1.4-fold higher normalized levels of plasma miR-132-3p than naïve rats (0.47 ± 0.14 vs. 0.33 ± 0.11, *p* < 0.05). TBI rats had 3.1-fold higher miR-132-3p levels than naïve rats (1.04 ± 0.43 vs. 0.33, *p* < 0.001) and 2.2-fold higher levels than sham-operated controls (1.04 vs. 0.47, *p* < 0.001).

#### 2.4.2. ROC Analysis

**Figure 4:**
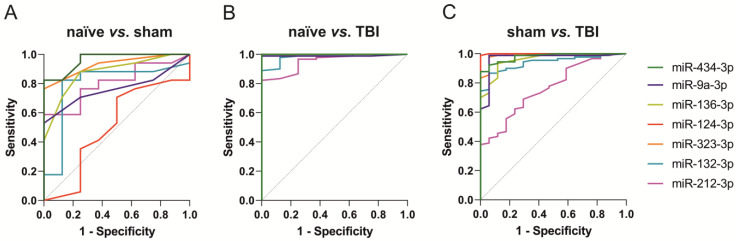
ROC analysis of plasma miRNA levels measured on D2 after TBI or sham operation. (**A**) Naïve vs. sham. Of 7 miRNAs, 6 (miR-434-3p, miR-9a-3p, miR-136-3p, miR-323-3p, miR-132-3p, and miR-212-3p) separated sham-operated controls (*n* = 17) from naïve rats (*n* = 8; *p* < 0.05). MiR-124-3p did not separate sham-operated controls from naïve animals (AUC 0.59, *p* > 0.05). See results for details. (**B**) TBI vs. naïve. All analyzed miRNAs separated rats with TBI (*n* = 90) from naïve animals (*n* = 8) with an AUC ≥ 0.90 (*p* < 0.001), indicating a very good performance as diagnostic TBI biomarkers. (**C**) TBI vs. sham operation. All analyzed miRNAs separated rats with TBI (*n* = 90) from sham-operated controls (*n* = 17). MiR-434-3p, miR-9a-3p, miR-136-3p, miR-323-3p, and miR-132-3p had AUC ≥ 0.90 (*p* < 0.001), whereas miR-212-3p had an AUC of 0.76 (*p* < 0.001). See results for details. Abbreviations: TBI, traumatic brain injury.

The ROC curves for miR-434-3p, miR-9a-3p, miR-136-3p, miR-323-3p, miR-124-3p, miR-212-3p, and miR-132-3p are presented in Figure 4. The AUC and optimal cut-off values for the normalized miRNA levels measured by ddPCR are summarized in Supplementary Table S7.

**miR-434-3p.** The miR-434-3p levels separated sham-operated controls from naïve rats (AUC 0.96, *p* < 0.001) with 82% sensitivity and 100% specificity (cut-off 0.22). TBI rats were separated from naïve rats (AUC 1.00, *p* < 0.001) with 100% sensitivity and 100% specificity (cut-off 0.31) and from sham-operated controls (AUC 0.98, *p* < 0.001) with 88% sensitivity and 100% specificity (cut-off 0.69).

**miR-9a-3p.** The miR-9a-3p levels separated sham-operated controls from naïve rats (AUC 0.76, *p* < 0.05) with 53% sensitivity and 100% specificity (cut-off 0.04). TBI rats were separated from naïve rats (AUC 0.99, *p* < 0.001) with 99% sensitivity and 100% specificity (cut-off 0.18) and from sham-operated controls (AUC 0.97, *p* < 0.001) with 99% sensitivity and 94% specificity (cut-off 0.18).

**miR-136-3p**. The miR-136-3p levels separated sham-operated controls from naïve rats (AUC 0.88, *p* < 0.01) with 88% sensitivity and 75% specificity (cut-off 0.03). TBI rats were separated from naïve rats (AUC 1.00, *p* < 0.001) with 99% sensitivity and 100% specificity (cut-off 0.05) and from sham-operated controls (AUC 0.96, *p* < 0.001) with 93% sensitivity and 88% specificity (cut-off 0.07).

**miR-323-3p.** The miR-323-3p levels separated sham-operated controls from naïve rats (AUC 0.93, *p* < 0.001) with 76% sensitivity and 100% specificity (cut-off 0.04). TBI rats were separated from naïve rats (AUC 1.00, *p* < 0.001) with 100% sensitivity and 100% specificity (cut-off 0.09) and from sham-operated controls (AUC 0.99, *p* < 0.001) with 98% sensitivity and 94% specificity (cut-off 0.12).

**miR-124-3p.** The miR-124-3p levels did not separate sham-operated controls from naïve rats (AUC 0.49, *p* > 0.05). The miR-124-3p levels separated TBI rats from naïve rats (AUC 1.00, *p* < 0.001, 100% sensitivity and 100% specificity, cut-off 0.20) and sham-operated controls (AUC 1.00, *p* < 0.001, 99% sensitivity and 100% specificity, cut-off 0.26).

**miR-212-3p.** The miR-212-3p levels separated sham-operated controls from naïve rats (AUC 0.81, *p* < 0.05) with 59% sensitivity and 100% specificity (cut-off 0.33). TBI rats were separated from naïve rats (AUC 0.95, *p* < 0.001) with 82% sensitivity and 100% specificity (cut-off 0.33) and from sham-operated controls (AUC 0.76, *p* < 0.001) with 69% sensitivity and 71% specificity (cut-off 0.39).

**miR-132-3p.** The miR-132-3p levels separated sham-operated controls from naïve rats (AUC 0.79, *p* < 0.05) with 82% sensitivity and 88% specificity (cut-off 0.39). TBI rats were separated from naïve animals (AUC 0.98, *p* < 0.001) with 89% sensitivity and 100% specificity (cut-off 0.60) and from sham-operated (AUC 0.94, *p* < 0.001) with 87% sensitivity and 94% specificity (cut-off 0.65).

### 2.5. Epilepsy Outcome

#### 2.5.1. D2 Plasma miRNA Levels

Next, we investigated whether plasma miRNA levels on D2 differentiated TBI rats with (TBI+) or without epilepsy (TBI−) or different epilepsy severities.

**Figure 5:**
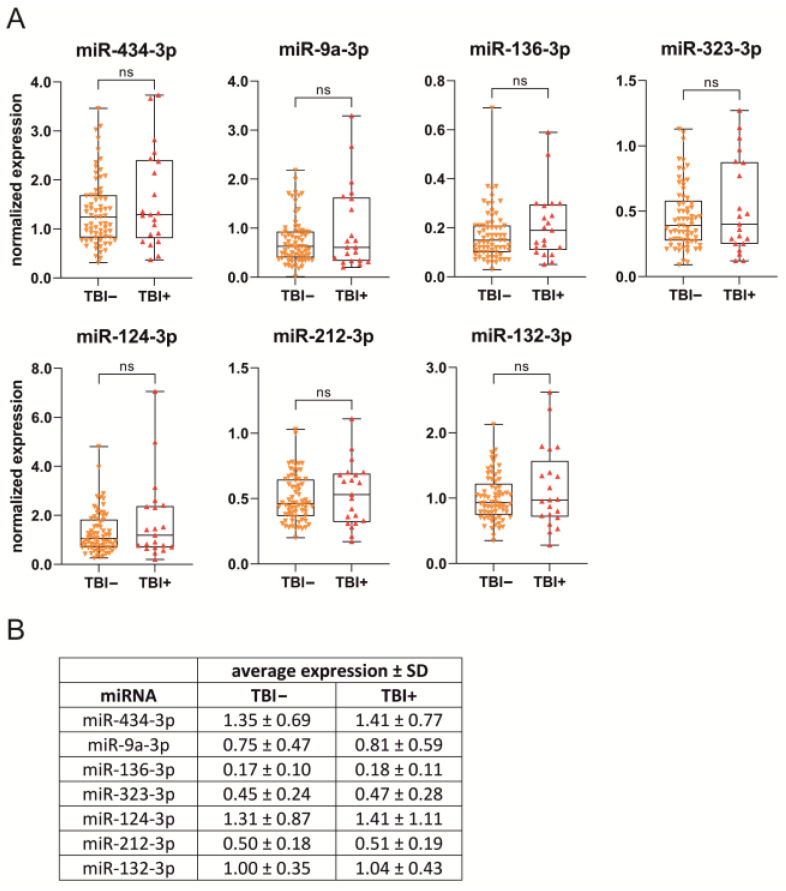
Normalized plasma miRNA levels on D2 and epilepsy outcome. (**A**) Box and whisker plots (whiskers: minimum and maximum; box: interquartile range; line: median) showing comparable plasma miRNA levels in the injured rats with (TBI+) and without (TBI−) epilepsy (Mann–Whitney U test, *p* > 0.05 for all miRNAs). (**B**) A table summarizing normalized miRNA expression levels (mean ± SD) in the TBI− and TBI+ groups. Abbreviations: ns, not significant; SD, standard deviation; TBI, traumatic brain injury.

***Epilepsy vs*. *no epilepsy***. Of the 90 TBI rats included in the miRNA analysis, 21 had epilepsy (TBI+) and 69 did not (TBI−). We detected no differences in the miRNA levels between the TBI+ and TBI− groups (*p* > 0.05 for all miRNAs) (Figure 5).

***Epilepsy severity–seizure frequency.*** To investigate possible differences in plasma miRNA levels within the TBI+ group depending on the severity of epilepsy, we categorized the 21 TBI+ animals based on two criteria: seizure frequency and occurrence of seizure clusters. 

**Figure 6:**
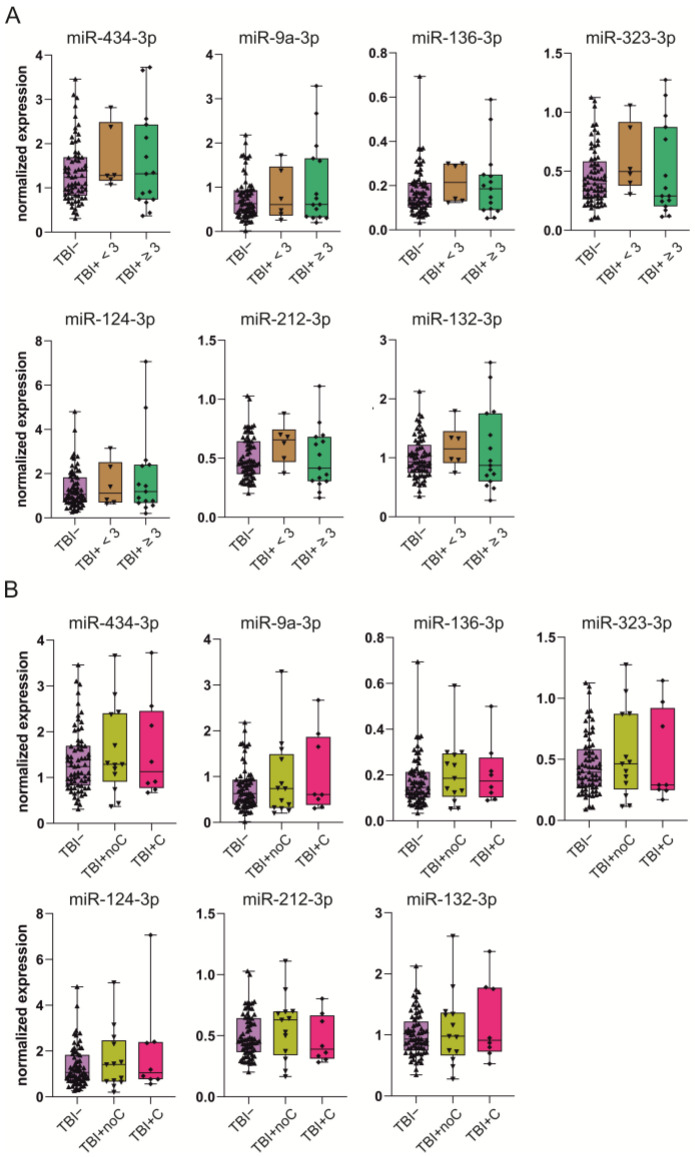
Normalized plasma miRNA levels on D2 and severity of epilepsy. (**A**) Rats with frequent (i.e., ≥3 late seizures per month) vs. less frequent seizures (<3 seizures per month). Box and whisker plots (whiskers: minimum and maximum; box: interquartile range; line: median) show that plasma miRNA levels on D2 in TBI+ animals with frequent (15/21) or less frequent late seizures (6/21) were comparable (Mann–Whitney U test, *p* > 0.05). In addition, the average miRNA levels were comparable when the TBI+ rats with frequent seizures were compared with TBI− animals or with all remaining TBI rats (TBI+ > 3 seizures vs. TBI− and TBI+ < 3 seizures; *p* > 0.05). (**B**) Rats with or without seizure clusters (i.e., ≥3 seizures within 24 h). Box and whisker plots summarize the levels of miRNAs in rats with epilepsy (TBI+) with seizure clusters (TBI+ C, 8/21) or without seizure clusters (TBI+ noC, 13/21). Plasma miRNAs on D2 did not differ between the rats with or without seizure clusters (Mann–Whitney U test, *p* > 0.05 for all miRNAs). In addition, plasma miRNA levels were comparable when the TBI+ rats with seizure clusters (severe epilepsy) were compared with TBI− rats or with all remaining TBI rats (TBI+ C vs. TBI− and TBI+ noC; TBI−, *n* = 69, *p* > 0.05). Abbreviations: TBI, traumatic brain injury; TBI−, TBI rats without epilepsy.

First, rats with epilepsy were divided according to whether the total number of seizures was ≥3 (*n* = 15) vs. <3 seizures (*n* = 6) during the 1-month vEEG monitoring. We detected no differences in the miRNA levels between the rats with ≥3 seizures or <3 seizures (*p* > 0.05 for all miRNAs) (Figure 6A). The total number of seizures did not correlate with any of the miRNAs (*p* > 0.05).

***Epilepsy severity–seizure clusters.*** For the analysis, rats with epilepsy were divided into two categories according to whether the animal had seizure clusters (8 yes, 13 no). A seizure cluster was defined as ≥3 seizures within 24 h. We detected no differences in different miRNA levels between the rats with or without seizure clusters (*p* > 0.05 for all miRNAs) (Figure 6B).

#### 2.5.2. ROC Analysis

The AUC and optimal cut-off values for the normalized miRNA levels measured by ddPCR are summarized in Supplementary Table S8.

**Figure 7:**
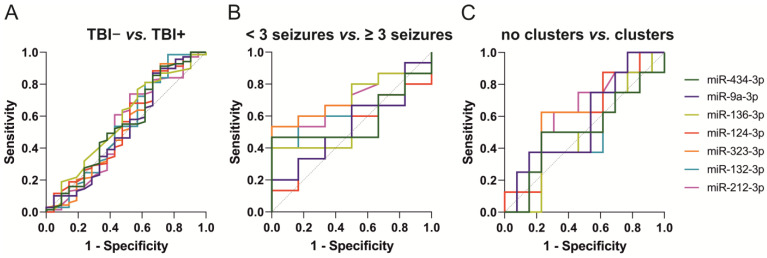
ROC analysis of plasma miRNA levels measured on D2 between epilepsy severity groups. None of the plasma miRNAs measured on D2 after traumatic brain injury (TBI) differentiated: (**A**) TBI rats with epilepsy (TBI+, *n* = 21) from rats without epilepsy (TBI−, *n* = 69); (**B**) TBI+ rats with ≥3 seizures (*n* = 15) from TBI+ rats with <3 seizures (*n* = 6); and (**C**) TBI+ rats with seizure clusters (*n* = 8) from TBI+ rats without seizure clusters (*n* = 13).

***Epilepsy* vs. *no epilepsy.*** None of the seven miRNAs distinguished TBI+ from TBI− rats on D2 (Figure 7A).

***Epilepsy severity–seizure frequency.*** None of the seven miRNAs distinguished rats with ≥3 or <3 seizures (Figure 7B).

***Epilepsy severity–seizure clusters.*** None of the seven miRNAs distinguished rats with or without clusters (Figure 7C).

#### 2.5.3. Glmnet Analysis

We then used elastic net regularized logistic regression (glmnet) to determine the optimal set of explanatory variables (i.e., miRNAs) to use in the logistic regression analysis to distinguish between TBI+ and TBI− animals, as well as to distinguish between the epilepsy severity groups.

***TBI+* vs. *TBI−.*** Glmnet analysis did not identify any combination of miRNAs (all coefficients zero in the majority of the cross-validation folds) that differentiated TBI+ rats (*n* = 21) from TBI− rats (*n* = 69).

**Figure 8:**
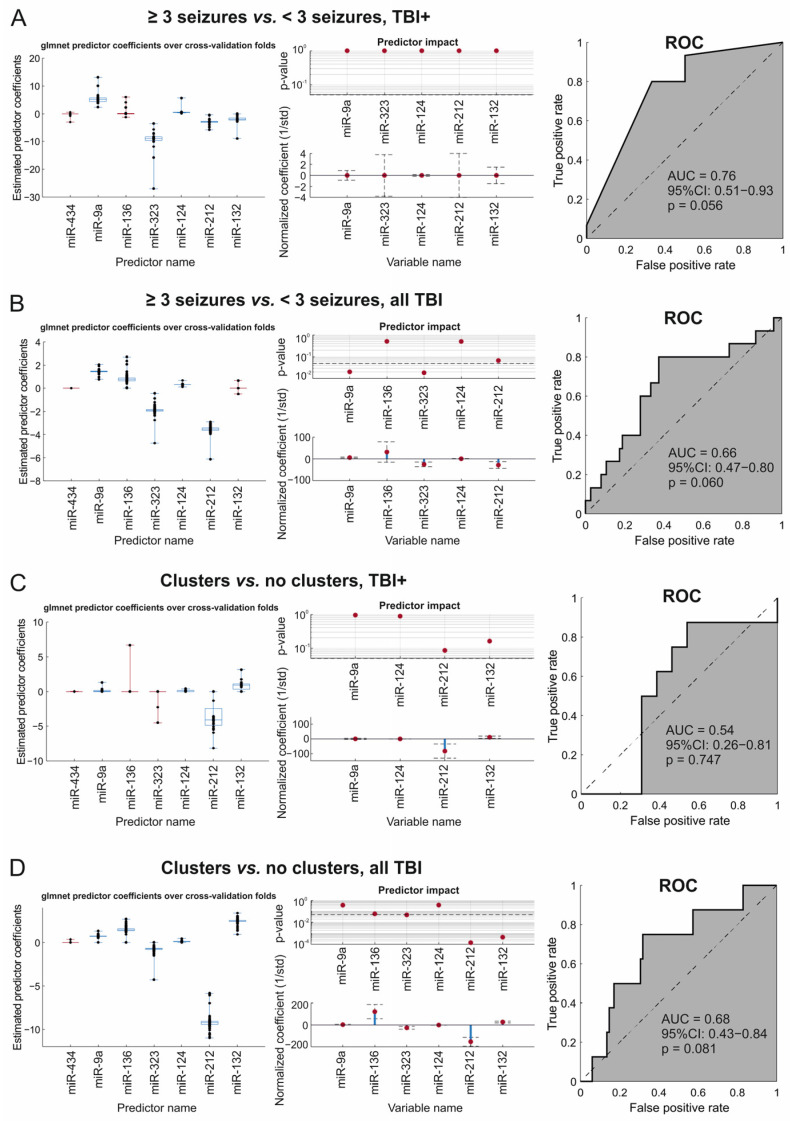
Elastic net regularized logistic regression (glmnet) analysis of plasma miRNA profile in epilepsy severity groups. (**A**) **Left panel**: Boxplots of predictor coefficients over cross-validation folds. The analysis defined miR-9a-3p, miR-323-3p, miR-124-3p, miR-212-3p, and miR-132-3p (blue boxplots) as the optimal set of miRNAs to separate TBI+ rats with ≥3 seizures/month (*n* = 15) from TBI+ rats with <3 seizures/month (*n* = 6). Due to having a coefficient of zero in most cross-validation folds, miR-434-3p and miR-136-3p were excluded (red boxplot). Center panel: *p*-value and normalized coefficient of each predictor in the standard logistic regression analysis. **Right panel**: Cross-validated ROC AUC was only 0.76 with a large confidence interval and *p* > 0.05. (**B**) The analysis defined miR-9a-3p, miR-136-3p, miR-323-3p, miR-124-3p, and miR-212-3p as the optimal set of miRNAs to separate TBI+ rats with ≥3 seizures/month (*n* = 15) from all remaining TBI rats (*n* = 75), but the ROC AUC was 0.66 with a large confidence interval and *p* > 0.05. (**C**) The analysis defined miR-9a-3p, miR-124-3p, miR-212-3p, and miR-132-3p as the optimal set of miRNAs to separate TBI+ rats with (*n* = 8) or without (*n* = 13) seizure clusters. The ROC AUC, however, was only 0.54 with a large confidence interval and a nonsignificant *p*-value (*p* > 0.05). (**D**) The analysis defined miR-9a-3p, miR-136-3p, miR-323-3p, miR-124-3p, miR-212-3p, and miR-132-3p as the optimal set of miRNAs to separate TBI+ rats with seizure clusters (*n* = 8) from all remaining TBI rats (*n* = 82). The ROC AUC was 0.68, however, with a larger confidence interval and *p* > 0.05. Abbreviations: AUC, area under the curve; CI, confidence interval; ROC, receiver operating characteristics; TBI, traumatic brain injury; TBI+, rats with epilepsy; TBI−, rats without epilepsy.

***≥3 seizures* vs. *<3 seizures.*** Glmnet analysis identified a combination of five miRNAs (miR-9a-3p, miR-323-3p, miR-124-3p, miR-212-3p, and miR-132-3p) as the optimal combination for differentiating TBI+ rats with ≥3 seizures (*n* = 15) from TBI+ rats with <3 seizures (*n* = 6). Logistic regression analysis using these predictors produced a cross-validated AUC of 0.76 (95% CI: 0.51–0.93, *p* = 0.056) (Figure 8A).

Next, we investigated the optimal combination for differentiating TBI+ rats with ≥3 seizures (*n* = 15) from all TBI rats (TBI+ with <3 seizures and TBI− rats combined, *n* = 75). Glmnet analysis identified a combination of five miRNAs (miR-9a-3p, miR-136-3p, miR-323-3p, miR-124-3p, and miR-212-3p) as the optimal set of miRNAs for differentiating the groups (AUC 0.66, 95% CI: 0.47–0.80, *p* = 0.060) (Figure 8B).

***Clusters vs*. *no clusters.*** Glmnet analysis identified miR-9a, miR-124-3p, miR-212-3p, and miR-132-3p as the optimal combination for differentiating TBI+ rats with seizure clusters (*n* = 8) from TBI+ rats without seizure clusters (*n* = 13) (Figure 8C). The AUC was low (0.54), however, and the confidence interval range large (95% CI: 0.26–0.81, *p* = 0.747).

Next, we investigated the optimal miRNA combination for differentiating TBI+ rats with seizure clusters (*n* = 8) from all TBI rats (TBI+ with no clusters and TBI− rats combined, *n* = 82). Glmnet analysis identified a combination of six miRNAs (miR-9a-3p, miR-136-3p, miR-323-3p, miR-124-3p, miR-212-3p, and miR-132-3p) as the optimal miRNA set for differentiating the groups (AUC 0.68, 95% CI: 0.43–0.84, *p* = 0.081) (Figure 8D).

### 2.6. Correlation of Acute Post-Injury Plasma miRNA Levels with Structural Outcome

To address whether the elevated plasma miRNAs report on the severity of cortical injury after TBI, we correlated the D2 miRNA levels with the severity of the lateral FPI-induced cortical lesion at acute and chronic time-points. For that, we measured: (a) the volume of abnormal cortical T_2_ in MRI on D2, D7, and D21 post-injury, and (b) the cortical lesion area in unfolded cortical maps on D182 post-injury.

#### 2.6.1. Correlation of miRNA Levels with Cortical Lesion Volume in MRI

**Figure 9:**
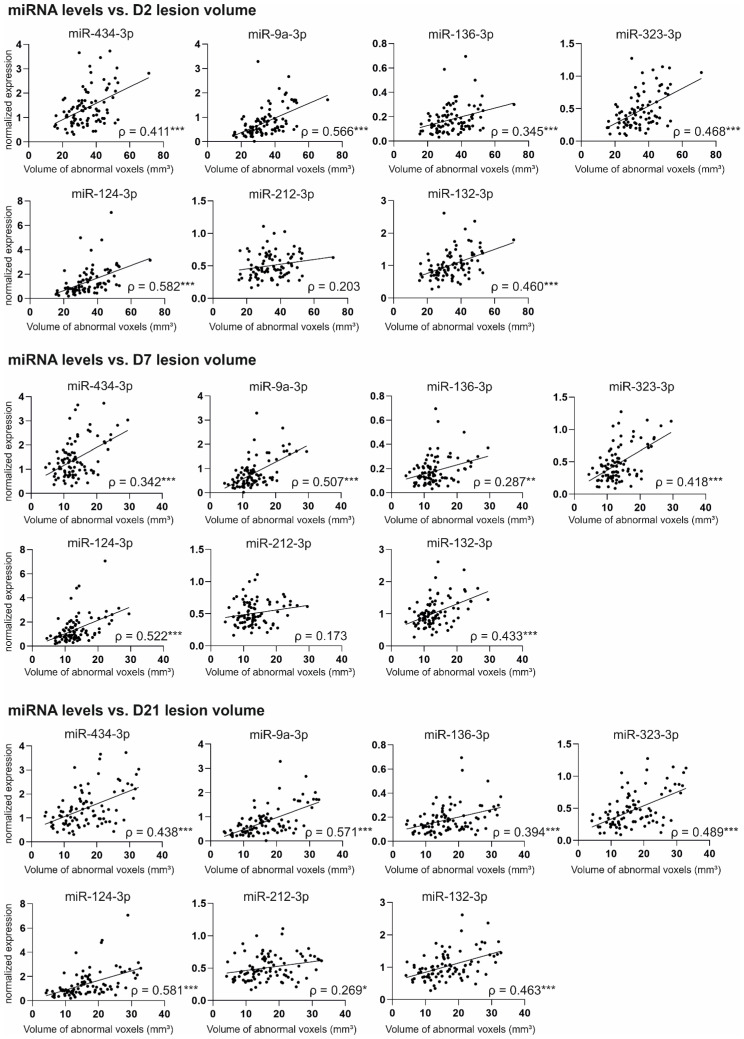
Spearman correlations (*ρ*) between normalized plasma miRNA levels on D2 and cortical lesion severity in quantitative T2 MRI on D2, D7, and D21 after TBI. Each dot represents 1 TBI animal (*n* = 89 for D2, *n* = 90 for D7 and D21). The higher the plasma miRNA levels on D2, the larger the lesion volume in MRI. The total volume of abnormal cortical T2 is shown on the *x*-axis and the normalized plasma miRNA level is shown on the *y*-axis. Note that the correlation coefficients were highest on D2 and D21. Statistical significance: *, *p* < 0.05; **, *p* < 0.01; ***, *p* < 0.001 (Spearman correlation). Abbreviations: D2, day 2 after TBI; D7, day 7 after TBI; D21, day 21 after TBI; TBI, traumatic brain injury.

The T_2_ MRI analysis for the whole EPITARGET cohort was previously presented by [20]. Quantitative T_2_ MRI data were available for all 90 TBI rats included in the plasma miRNA analysis, except for one animal missing D2 MRI data. Consequently, the animal numbers were 89 for D2, 90 for D7, and 90 for D21. Correlation plots for normalized miRNA levels and T_2_ MRI data are summarized in Figure 9.

**miR-434-3p**. The higher the plasma levels of miR-434-3p on D2, the larger the volume of the ipsilateral cortical T_2_ abnormality on D2 (*ρ* = 0.411, *p* < 0.001), on D7 (*ρ* = 0.342, *p* < 0.001), and on D21 (*ρ* = 0.438, *p* < 0.001).

**miR-9a-3p**. The higher the plasma levels of miR-9a-3p on D2, the larger the volume of the ipsilateral cortical T_2_ abnormality on D2 (*ρ* = 0.566, *p* < 0.001), on D7 (*ρ* = 0.507, *p* < 0.001), and on D21 (*ρ* = 0.571, *p* < 0.001).

**miR-136-3p.** The higher the plasma levels of miR-136-3p on D2, the larger the volume of the ipsilateral cortical T_2_ abnormality on D2 (*ρ* = 0.345, *p* < 0.001), on D7 (*ρ* = 0.287, *p* < 0.01), and on D21 (*ρ* = 0.394, *p* < 0.001).

**miR-323-3p.** The higher the plasma levels of miR-323-3p on D2, the larger the volume of the ipsilateral cortical T_2_ abnormality on D2 (*ρ* = 0.468, *p* < 0.001), on D7 (*ρ* = 0.418, *p* < 0.001), and on D21 (*ρ* = 0.489, *p* < 0.001).

**miR-124-3p**. The higher the plasma levels of miR-124-3p on D2, the larger the volume of the ipsilateral cortical T_2_ abnormality on D2 (*ρ* = 0.582, *p* < 0.001), on D7 (*ρ* = 0.522, *p* < 0.001), and on D21 (*ρ* = 0.581, *p* < 0.001).

**miR-212-3p**. The higher the plasma levels of miR-212-3p on D2, the larger the volume of the ipsilateral cortical T_2_ abnormality on D21 (*ρ* = 0.269, *p* < 0.05) but not on D2 or D7 (*p* > 0.05).

**miR-132-3p**. The higher the plasma levels of miR-132-3p on D2, the larger the volume of the ipsilateral cortical T_2_ abnormality on D2 (*ρ* = 0.460, *p* < 0.001), on D7 (*ρ* = 0.433, *p*<0.001), and on D21 (*ρ* = 0.463, *p* < 0.001).

**miR-28-3p**. We detected no correlation between the plasma miR-28-3p levels and the volume of the ipsilateral cortical T_2_ abnormality at any time-point (*p* > 0.05).

#### 2.6.2. Correlation of miRNA Levels with Cortical Lesion Area in Unfolded Maps

**Figure 10:**
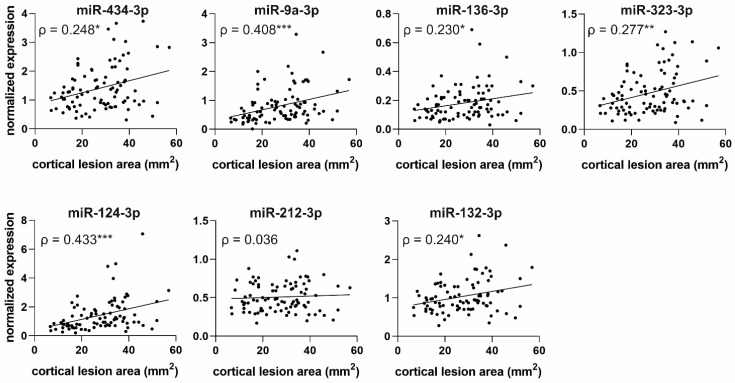
Spearman correlations (*ρ*) between normalized plasma miRNA levels on D2 and cortical lesion area in unfolded cortical maps on D182 after TBI. The greater the normalized plasma levels of miR-9a-3p and miR-124-3p on D2 after TBI (*n* = 90), the greater the cortical lesion area on D182 (ρ > 0.4). In addition, elevated normalized plasma miR-434-3p, miR-136-3p, miR-323-3p, and miR-132-3p levels were associated with a greater cortical lesion area 6 months later (*ρ* > 0.2). No correlation was detected between miR-212-3p level and cortical lesion area (*ρ* = 0.036, *p* > 0.05). Statistical significance: *, *p* < 0.05; **, *p* < 0.01; ***, *p* < 0.001 (Spearman correlation).

Unfolded cortical maps were created for all 90 TBI rats included in the miRNA analysis. Correlation plots for normalized miRNA levels on D2 and cortical lesion area on D182 are presented in Figure 10. 

Correlation analysis showed that the greater the normalized plasma levels of all miRNAs on D2, the larger the cortical lesion area on D182. The only exception was miR-212-3p (*p* > 0.05). The miRNAs most strongly associated with the extent of cortical lesion area were miR-124-3p (*ρ* = 0.433, *p* < 0.001) and miR-9a-3p (*ρ* = 0.408, *p* < 0.001), followed by miR-323-3p (*ρ* = 0.277, *p* < 0.01), miR-434-3p (*ρ* = 0.248, *p* < 0.05), miR-132-3p (*ρ* = 0.240, *p* < 0.05), and miR-136-3p (*ρ* = 0.230, *p* < 0.05). We detected no correlation between the endogenous control miR-28-3p levels and cortical lesion area (*p* > 0.05).

### 2.7. MiRNA Target Analysis

Ingenuity pathway analysis (IPA) was used to search for the predicted mRNA targets of the validated miRNAs. We also conducted a pathway analysis to investigate the canonical pathways associated with the target genes. 

IPA found 1 target for miR-434-3p, 89 targets for miR-9a-3p, 27 targets for miR-136-3p, 29 targets for miR-323-3p, 460 targets for miR-124-3p, and 91 targets for miR-132-3p/miR-212-3p. The targets are visualized in Supplementary Figure S9A. Canonical pathway analysis of the predicted target genes revealed different top canonical pathways for each miRNA, except for miR-434-3p for which no data was available (Supplementary Figure S9B). The top canonical pathways included pathways associated with cancer, inflammation, and immune cell function.

## 3. Discussion

The present study investigated whether acute alterations in the circulating plasma miRNA expression levels after experimental TBI can be used as prognostic biomarkers for the evolution of cortical damage and epileptogenesis after TBI. Four major findings were revealed. First, small RNA sequencing revealed 45 differentially expressed miRNAs between the TBI and sham-operated controls on D2 after injury. Second, craniotomy without TBI induction resulted in miRNA upregulation compared with naïve rats. Third, the higher the plasma miRNA levels at the acute post-TBI time-point, the larger the cortical lesion within both the first 3 weeks and 6 months after injury. Fourth, D2 plasma miRNA levels did not predict post-traumatic epileptogenesis or epilepsy severity.

### 3.1. Acute Post-Injury Regulation of Circulating Neuron-Enriched miRNAs Is Clear but Short-Lasting

We investigated TBI-induced alterations in plasma miRNA expression at acute (D2) and subacute (D9) time-points after TBI. Small RNA sequencing revealed that the plasma miRNA expression profile separated TBI from sham rats on D2 but not on D9. The two time-points had no common upregulated miRNAs and shared only three downregulated miRNAs, suggesting that most of the miRNAs with altered expression on D2 returned to baseline levels by D9 after injury. Consequently, we focused our analysis on validating differentially expressed miRNAs found on D2 after TBI. The miRNAs selected for validation were brain-enriched and had at least a 2-fold (log2FC ≥ 1) difference between the TBI and sham groups and a CPM value ≥30. 

Using ddPCR, we showed increased expression of seven miRNAs (miR-434-3p, miR-9a-3p, miR-136-3p, miR-323-3p, miR-124-3p, miR-212-3p, and miR-132-3p) in the rat plasma on D2 after TBI compared with sham-operated (craniotomy) controls. All analyzed miRNAs are primarily expressed in neurons [10,21,22,23,24,25,26,27,28]. The greatest fold changes were observed for miR-9a-3p (22-fold) and miR-124-3p (18-fold). The dysregulation of miR-323-3p (7-fold) as well as miR-136-3p and miR-434-3p (around 4-fold for both) was also clear. Upregulation of miR-132-3p and miR-212-3p was approximately 2-fold and 1.5-fold, respectively, after TBI.

Our study also included naïve controls that did not undergo any surgery. Interestingly, the fold changes in the plasma miRNA expression were greater when TBI rats were compared with naïve rats than when they were compared with sham-operated controls. The largest fold change was observed for miR-9a-3p (41-fold), followed by miR-323-3p (31-fold) and miR-124-3p (21-fold). Upregulation of miR-434-3p and miR-136-3p was approximately 11-fold, and upregulation of miR-132-3p and miR-212-3p was approximately 3-fold and 2-fold, respectively.

We previously reported upregulation of miR-434-3p, miR-9a-3p, miR-136-3p [18], and miR-124-3p [29] in the plasma on D2 after LFPI-induced TBI. The fold changes of miR-9a-3p, miR-434-3p, and miR-136-3p levels after TBI in the present study were comparable to our previous data [18]. The 18-fold increase in miR-124-3p levels on D2 in TBI rats compared with sham-operated controls, however, was clearly higher than the 1.4-fold increase reported by [29]. This may relate to slight differences in the ddPCR analysis methods. In the present study, we used ddPCR with EvaGreen chemistry, whereas the study by Vuokila et al. [29] used ddPCR with TaqMan chemistry. Interestingly, Wang et al. [30] reported a >2-fold increase in exosomal miR-124-3p levels in the rat plasma at 24 h after weight-drop–induced TBI, indicating that miR-124-3p levels report on brain injury induced by different mechanisms. To the best of our knowledge, the levels of miR-323-3p, miR-132-3p, and miR-212-3p in blood circulation have not been previously investigated in TBI models.

We used ROC analysis to assess the performance of the seven validated miRNAs as diagnostic biomarkers for TBI. We found that acutely increased levels of all seven miRNAs distinguished TBI rats from naïve rats with an ROC AUC ≥ 0.90. All seven miRNAs also distinguished TBI from sham controls and 6 of 7 had an ROC AUC ≥ 0.90. Taken together, the validated circulating miRNAs demonstrate excellent performance as diagnostic biomarkers for brain injury. 

We next investigated whether the miRNAs detected in preclinical models are also dysregulated after human TBI. Some of the miRNAs analyzed in our study were previously investigated in blood samples collected from TBI patients [18,31,32,33]. O’Connell et al. reported a 4-fold increase in the serum miR-9-3p levels and a 3-fold increase in the serum miR-124-3p levels in TBI patients at hospital admission (≤24 h after injury) [31]. The patients included in the study had variable injury severities with a mean Glasgow Coma Score (GSC) of 10.8 ± 2.5. Schindler et al. [33] detected miR-124-3p in the blood of patients with severe TBI within 6 h after injury; the fold change compared with controls, however, was <2. In our previous study [18], we detected a 9-fold increase in miR-9-3p levels in plasma collected from severe TBI patients (≤48 h after injury). In addition, we detected elevated plasma levels of miR-9-3p and miR-136-3p in a subpopulation of mild TBI patients with high plasma S100B levels.

To the best of our knowledge, there is only one study on blood miR-132-3p levels in TBI patients [32]. That study used a microarray and reported a 2-fold increase in serum miR-132-3p levels at 12 h and 48 h post-injury compared with the 2-h post-injury time-point. Interestingly, they did not detect an increase in miR-132-3p levels at 24 h post-injury. 

Taken together, we report the upregulation of seven neuronally enriched plasma miRNAs on D2 in a clinically relevant experimental model of closed head injury. Of these, miR-434-3p is rodent-specific and not detected in humans [18]. Two of the miRNAs, however, miR-323-3p and miR-212-3p, have not yet been analyzed in humans, and thus present novel upregulated candidate plasma biomarkers to be validated in TBI patients. 

### 3.2. Plasma miRNA Levels Detect Mild Injury Caused by Craniotomy during Sham Operation

Craniotomy can induce structural and functional damage to the underlying brain [34]. In addition, craniotomy results in mild T_2_ relaxation abnormalities in MRI and increased levels of circulating neurofilament light, a protein marker of axonal injury [35].

In the present study, we detected an increase in 6 of 7 miRNAs (all except miR-124-3p) in sham-operated controls on D2 after surgery compared with naïve rats. The six upregulated miRNAs distinguished the sham-operated rats from naïve rats in the ROC analysis. In contrast to the other miRNAs, the plasma levels of miR-124-3p were unaltered in the sham-operated control group compared with naïve rats, and miR-124-3p levels did not separate sham from naïve animals in the ROC analysis. This suggests that the release of miR-124-3p into blood circulation requires an impact stronger than craniotomy, whereas miR-434-3p, miR-9a-3p, miR-323-3p, miR-136-3p, miR-212-3p, and miR-132-3p appear to be sensitive indicators of injury induced by craniotomy. All of the investigated miRNAs are neuronally enriched, suggesting that their release into the plasma results from neuronal injury rather than injury to other tissues, such as bone or skin. As there are limited data available on the expression of these miRNAs in other tissues, or on the impact of TBI on miRNA expression in organs other than the brain, a peripheral contribution cannot be completely excluded.

The ability to distinguish sham-operated controls from naïve rats makes miR-434-3p, miR-9a-3p, miR-323-3p, miR-136-3p, miR-212-3p, and miR-132-3p potential biomarkers of mild brain injury. In our earlier study with a low number of TBI patients [18], miR-9-3p and miR-136-3p did not differentiate mild TBI patients from controls in the ROC analysis, and the rodent-specific miR-434-3p was not detected in humans. On the other hand, the remaining three miRNAs, miR-323-3p, miR-212-3p, and miR-132-3p, have not been previously investigated after mild TBI. 

Taken together, our results indicate that craniotomy can cause injury to the brain, leading to a detectable increase of neuron-enriched miRNAs in plasma. Of the analyzed miRNAs, miR-323-3p, miR-212-3p, and miR-132-3p in particular have potential as sensitive markers of mild brain injury and warrant further preclinical and clinical studies.

### 3.3. Functions of the Analyzed miRNAs

The cellular and molecular aftermath after TBI is proposed to include multiple sequentially progressing pathologies, such as apoptosis, neurodegeneration, astrogliosis, axonal injury, and demyelination [36]. We hypothesized that the function of the upregulated miRNAs will report on some of these ongoing pathologies on D2 after injury. Consequently, we performed a literature search to obtain an overview of the functions of the analyzed miRNAs in healthy brain and the impact of neurodegenerative diseases on their expression in the blood and brain.

On D2 after TBI, the most prominent upregulation was observed for miR-9a-3p (22-fold increase compared with sham). MiR-9 is highly enriched in the vertebrate developing and mature nervous system [37]. It functions in embryonic neurogenesis and regulates neural progenitor proliferation [23,37]. Inhibition of miR-9-3p in mature neurons impairs hippocampal long-term potentiation in mice, supporting its role in regulating synaptic plasticity and memory [38]. As noted earlier, a TBI-induced increase in miR-9-3p levels in the blood is reported in both TBI animal models and patients [18]. The release of miR-9 from the brain to the blood or CSF, however, is not specific to TBI. Several studies report elevated blood or CSF miR-9 levels in stroke patients [39,40,41]. In addition, increased plasma miR-9 levels are reported to associate with an unfavorable neurologic outcome after cardiac arrest [42]. Taken together, these studies indicate that miR-9 is a general marker of brain injury.

The second largest increase after TBI was observed for miR-124-3p (18-fold increase compared with sham). MiR-124 is specifically expressed in the brain of both rodents and humans [10,22,25], and it is the most abundant miRNA in the brain. MiR-124 functions in embryonic neurogenesis [23] and plays an important role in differentiating progenitor cells to mature neurons [24]. Brain injury induces an increase in circulating miR-124 levels at an acute post-TBI time-point in both animal models [29,30] and TBI patients [31]. Similar to miR-9, miR-124 appears to be a general marker of brain injury. In addition to TBI, alterations in circulating miR-124 are reported at an acute time-point in ischemic and hemorrhagic stroke patients [39,41,43,44].

The third largest increase after TBI was observed for miR-323-3p (7-fold increase compared with sham). MiR-323 is neuronally enriched [21,28]. To the best of our knowledge, the expression of miR-323 in the blood or brain has not been previously investigated in TBI models or TBI patients. Instead, miR-323 expression in plasma is reported to differentiate human subjects with mild cognitive impairment from age-matched controls [45]. One study reported increased plasma miR-323 expression in genetic Parkinson’s disease [46], but the results were not validated in a subsequent study [47].

Plasma miR-136-3p levels showed a 4.4-fold increase after lateral FPI. MiR-136 is specifically enriched in neurons [21,28]. Expression outside of the central nervous system is also reported. For example, Chen et al. [48] measured miR-136-3p in cultured human umbilical vein endothelial cells and bone mesenchymal stem cells. Kitahara et al. [49] detected upregulation of miR-136-3p in rat ovaries together with miR-434 and miR-132 following the administration of human chorionic gonadotropin. Because our study included only male rats, the possible expression of these miRNAs in the rat ovaries does not affect our results. As mentioned above, we previously reported increased plasma miR-136-3p levels on D2 after injury in a rat model of TBI [18]. In addition, increased miR-136-3p levels are detected in the plasma [46,47] and in CSF exosomes [50] of patients with Parkinson’s disease compared with healthy controls.

Plasma miR-434-3p showed a 4.2-fold increase on D2 after TBI. MiR-434 is a rodent-specific miRNA (miRBase, accessed on 9 September 2022). Although Jovičić et al. [28] showed that miR-434 is specifically expressed in rat cortical neurons, a few studies have also detected miR-434-3p in the skeletal muscle of rats and mice [51,52,53]. In neurons, miR-434-3p is suggested to be involved in regulating stress-induced transcripts [54]. A study with weight-drop-induced mild TBI detected upregulation of miR-434-3p in mouse plasma at 3 h post-injury [55]. As mentioned above, we previously reported increased miR-434-3p levels in rat plasma on D2 after mild TBI [18].

MiR-212 and miR-132 are closely related and located in the same miRNA cluster [56]. They are highly conserved among vertebrates. MiR-212 and miR-132 are enriched in neurons [21,28,57] but are also detected in other cells, such as immune cells [58,59], rat vascular smooth muscle cells [60], insulin-secreting β-cells [61], and several cancer types [56]. Importantly, miR-132 is detected in astrocytes and microglia in both experimental (rat) and human temporal lobe epilepsy (TLE) [62]. In the rat brain, miR-212 and miR-132 are reported to be especially enriched in the neurons of forebrain regions and less abundantly in the cerebellum [26]. Within the normal mouse cerebrum, miR-132 and miR-212 are most abundantly expressed in the frontal cortex compared with the hippocampus [27]. In neurons, miR-212 and miR-132 have important roles in regulating neurite outgrowth, dendrite maturation, and synaptic plasticity [57,63,64,65,66]. Knock-out of miR-132 and/or miR-212 in mice is linked to cognitive deficits [67,68]. MiR-132 is also involved in circadian clock function [69].

Studies on miR-212 and miR-132 in TBI are scarce. As mentioned above, there is only one study on blood miR-132-3p levels in TBI patients [32]. There are, however, a couple of studies on miR-212 and miR-132 in Alzheimer’s disease. One study reported downregulation of miR-212 and miR-132 in the temporal cortex of Alzheimer’s disease patients [70]. Another study found that reduced plasma exosomal miR-212 and miR-132 levels differentiated patients with Alzheimer’s disease from cognitively non-impaired controls [71].

In addition to a literature search, we used ingenuity pathway analysis (IPA) to determine the mRNA targets of the validated injury effect miRNAs, particularly whether there were any shared targets between the different miRNAs included in our analysis. In addition, we investigated the canonical pathways involving the target genes. The analysis revealed only a few common targets between the investigated miRNAs, and none of them were shared by more than two miRNAs, suggesting that the plasma-upregulated miRNAs report on the regulation of different pathways. There was also a large variation in the number of predicted targets. More than 400 targets were found for miR-124-3p, whereas only one target was found for miR-434-3p. As we noticed while conducting the literature search, the amount of data available on different miRNAs varies remarkably. This results in a large disparity in the amount of available miRNA target data in IPA, which can cause bias in the target and pathway analyses.

Taken together, all of the miRNAs validated in our study are brain-enriched, and alterations in their expression were previously detected in brain injury or in neurodegenerative diseases. Some of the miRNAs, such as miR-124 and miR-212/miR-132, have been studied more extensively than others, whereas the functions of, for example, miR-323, miR-136, and miR-434 in the brain remain unclear.

### 3.4. Acute Elevation of Plasma miRNA Levels Does Not Predict the Development of PTE

Several previous studies reported alterations in circulating miRNA expression levels in experimental models of epileptogenesis induced by electrical stimulation or chemoconvulsants and in epilepsy patients [15,72,73]. To the best of our knowledge, there are no prior studies on circulating miRNA expression in PTE models or in patients with TBI. The present study is the first to investigate plasma miRNAs in a model of PTE.

During the 6-month follow-up of the EPITARGET study, 25% of the TBI rats developed PTE. We searched for differentially expressed circulating miRNAs at an acute post-TBI time-point between rats that did (TBI+) or did not (TBI−) develop epilepsy, but differential expression analysis of the small RNA-seq data yielded no differentially expressed miRNAs between the groups on D2 or D9 after TBI. As shown in the Venn diagrams, on both D2 and D9, small RNA sequencing revealed 16 (D2) and 22 (D9) miRNAs detected in TBI+ but not in TBI− or sham animals. These miRNAs, however, typically had only 1–3 raw reads in one sample and no reads in the others. Therefore, they were not considered for further analysis.

Because previous studies demonstrated that the risk of PTE increases with injury severity [1], we investigated whether the acute post-TBI increase in plasma miRNAs predicted the development of epilepsy. We compared the levels of miR-434-3p, miR-9a-3p, miR-136-3p, miR-323-3p, miR-124-3p, miR-212-3p, and miR-132-3p on D2 between the TBI+ and TBI−rats. Disappointingly, no differences were detected.

We further investigated whether the miRNA levels on D2 differed between TBI+ rats with different epilepsy severities, using the total number of seizures and the presence of seizure clusters as markers of epilepsy severity. No differences were detected, however, between the rats with milder or severe PTE. 

Finally, we used elastic net regularized logistic regression (glmnet) to investigate whether a combination of miRNAs rather than a single miRNA could differentiate the TBI+/TBI− and epilepsy severity groups. Our analysis revealed no miRNA sets for TBI+/TBI− differentiation. For the epilepsy severity groups, the analysis did identify optimal sets of miRNAs to differentiate rats with milder or severe PTE but the findings were not statistically significant. It should be noted that the analyses included only 21 TBI+ rats and, consequently, the number of animals in the different epilepsy severity groups was small.

From the seven miRNAs investigated in our study, miR-132 and miR-212 are the most extensively studied in the context of epilepsy. Several studies report increased levels of miR-132 and/or miR-212 in the brain tissue of animal models of epileptogenesis induced with status epilepticus (SE). Nudelman et al. [74] observed a 50% increase in miR-132 levels in RT-qPCR analysis of mouse hippocampus at 8 h after pilocarpine injection. Bot et al. [75] reported a 2-fold upregulation of miR-132-3p and miR-212-3p in a miRNA array analysis of the rat dentate gyrus at 7 d after electrical amygdala stimulation-induced SE. Gorter et al. [76] also used a miRNA array and detected upregulation of miR-132 and miR-212 in the rat dentate gyrus at 1 day, 1 week, and 3 months after SE induced by electrical stimulation of the hippocampus by a stimulation electrode implanted in the angular bundle. Guo et al. [77] found a ~2-fold increase in brain miR-132 expression in the RT-qPCR analysis at 24 h and 7 d after lithium-pilocarpine-induced SE in rats. Similarly, Korotkov et al. [62] observed a 2-fold increase in miR-132 expression in the rat dentate gyrus at 1 d after SE induced by tetanic stimulation of the hippocampus. In contrast to other studies, however, they detected no differences in miR-132 expression at 1 week after SE. A recent study by Bencurova et al. [78] reported a 2-fold upregulation of miR-132-3p and a 3-fold upregulation of miR-212-3p in next-generation sequencing of rat hippocampal tissue 24 h after pilocarpine-induced SE. In addition, Venø et al. [79] reported upregulation of miR-132-3p and miR-212-3p in next-generation sequencing of hippocampal tissue in three different models of TLE, including SE induced by intra-amygdala kainic acid, systemic pilocarpine, or perforant path stimulation. They also reported that treatment with an miR-212-3p antagomir did not reduce seizure burden or neuronal damage after SE in the intra-amygdala kainic acid model. Treatment with an miR-132-3p antagomir was not tested. In contrast, Jimenez-Mateos et al. [80] observed a 5-fold increase in miR-132 levels in the CA3 region of the mouse hippocampus at 24 h after SE induction and reported that treatment with an miR-132 antagomir led to less apoptotic cells and more surviving neurons. There are also several studies on miR-132 and/or miR-212 in epilepsy patients, that is, at later stages of epileptogenesis, after the diagnosis of epilepsy and the occurrence of unprovoked seizures. A study by Peng et al. [81] found a 2-fold upregulation of miR-132 in hippocampal tissues of children with mesial TLE. Korotkov et al. [62] observed a ~3-fold higher expression of miR-132 in patients with TLE and hippocampal sclerosis compared with controls. In contrast to other studies, Guo et al. [77] reported a ~50% reduction in miR-132 expression in the temporal neocortex of TLE patients compared with controls, which could relate to neuronal loss and glial cell proliferation due to long-term effects of epilepsy. Recently, one study reported post-ictal elevation in plasma miR-132 levels at 2 h after seizure onset in patients with epilepsy [82]. Most of the patients (75%) had generalized tonic-clonic seizures, whereas the rest had simple or complex partial seizures. The study reported that the longer the disease course and the longer duration of seizures, the greater the increase in plasma miR-132 levels. Interestingly, opposite to miR-132, studies reported the downregulation of miR-212 in epilepsy patients. Haenisch et al. [83] found a 33% decrease in miR-212-3p expression in the hippocampus compared with the temporal neocortex in patients with mesial TLE. Another study reported a 50% decrease in miR-212 levels in both the serum and hippocampus of TLE patients [84]. In the present study, miR-124-3p was one of the miRNAs with the largest increase in the plasma after TBI, and miR-124 is a general marker of brain injury. Several studies on animal models report alterations in miR-124 expression in the brain tissue following the induction of SE. Vuokila et al. [85] observed a slight downregulation of miR-124 in the rat dentate gyrus at 7 d after SE in the electrical amygdala stimulation model. Ambrogini et al. [86] observed a trend toward decreased miR-124 levels in hippocampal homogenates and serum of rats 15 d after kainic-acid-induced SE, but the difference was not statistically significant compared with non-epileptic controls. Brennan et al. [87] observed a sharp decrease in miR-124 levels in rat hippocampus at 1.5–48 h after kainic acid-induced SE. The miR-124 levels were lowest 48 h after SE, ~10% of that in controls. The study also found that treatment of rats with miR-124 agomirs promoted inflammation and therefore suggested that reduction of miR-124 levels in the brain after SE may be an adaptive response to control excess inflammation. Interestingly, in contrast to other SE induction methods, SE induction by pilocarpine appears to have the opposite effect on miR-124 levels in the brain. Hu et al. [88] observed upregulation of miR-124 in rat hippocampus 24 h after lithium-pilocarpine-induced SE. Peng et al. [81] reported upregulation of miR-124 in the hippocampus of immature mesial TLE model rats 2 h and 8 weeks after pilocarpine-induced SE. For the remaining miRNAs analyzed in the present study (miR-9, miR-323, miR-434, and miR-136), only a few studies exist on their expression in the context of epilepsy. Brennan et al. [87] found no difference in rat hippocampal miR-9 levels following kainic-acid-induced SE. Jimenez-Mateos et al. [80] observed a 22-fold increase in miR-323 levels in the miRNA screening of mouse hippocampus at 24 h after intra-amygdalar kainic-acid-induced SE, but they did not validate the observation by PCR. Chen et al. [89] reported a 4-fold upregulation of miR-434-3p in the blood of mice 24 h after pilocarpine-induced SE. A recent study reported the reduced expression of miR-136 in the hippocampus of rats with pilocarpine-induced TLE at 7 d after SE induction [90]. They also showed that overexpression of miR-136 reduced the number and duration of seizures and the levels of pro-inflammatory cytokines and increased the number of neurons in the hippocampus, suggesting that miR-136 has neuroprotective effects. Taken together, our results showed that elevated plasma levels of the seven neuronally enriched miRNAs at an acute post-TBI time-point did not reach statistical power as prognostic biomarkers for post-traumatic epileptogenesis or epilepsy severity. This was unexpected as elevated brain levels of miR-132 and miR-212, in particular, within the acute time window assessed in the present study, are linked to epileptogenesis in chemoconvulsant and electrical-stimulation-induced epileptogenesis models. The most apparent explanation is the etiology specificity of the spatiotemporal evolution of brain pathology after epileptogenic insults and associated miRNA expression. 

### 3.5. Increased Plasma miRNA Levels at an Acute Post-TBI Time-Point Correlate with a Larger Cortical Lesion Area at Acute, Subacute, and Chronic Time-Points

One of our aims was to investigate whether circulating miRNA levels at an acute post-TBI time-point reflect injury severity in the brain and could be used to predict progression of the cortical pathology at later time-points.

During the first 3 weeks after TBI, we assessed the cortical pathology of the rats with T_2_ relaxation MRI on D2, D7, and D21 after the injury. The method detects edema as increased T_2_ and post-impact hemorrhage as decreased T_2_ [20]. We determined the cortical lesion volume from the total volume of imaging voxels with abnormal T_2_ values. We demonstrated that with 6 of 7 validated miRNAs, increased plasma miRNA levels on D2 after TBI correlated with a larger lesion volume in MRI at all three time-points. In addition to T_2_ MRI conducted at acute and subacute time-points, we assessed the cortical lesion area 6 months after TBI with histologic analysis. The analysis revealed a correlation between elevated acute plasma miRNA levels and a larger lesion area at the chronic time-point, although the correlations were slightly weaker than in the MRI analysis at earlier time-points.

In both MRI and histologic analysis, neuronally enriched miR-124 and miR-9 exhibited the strongest correlation with cortical pathology. We previously demonstrated that TBI causes the downregulation of miR-124-3p in the perilesional cortex in both rats and humans [29]. In the same study, we demonstrated that elevated plasma miR-124 levels correlate with a larger lesion area in MRI 2 months after LFPI-induced TBI [29].

The correlation of circulating miR-124 or miR-9 levels with cortical pathology is not limited to TBI. Several studies investigated blood miR-124 levels in stroke patients. Leung et al. [43] observed higher plasma miR-124 levels within <24 h after onset of symptoms in hemorrhagic stroke patients than in ischemic stroke patients or controls. Rainer et al. [91] reported that the higher the plasma miR-124-3p concentrations measured ≤24 h from stroke onset, the larger the lesion volume and the worse the stroke outcome. Ji et al. [39] detected increased miR-124 and miR-9 levels in exosomes isolated from the serum of acute ischemic stroke patients (average 16.5 h after stroke), and they reported that higher exosomal miR-124 and miR-9 levels correlated with larger infarct volume and worse outcome. Increased exosomal miR-124 and miR-9 levels in the serum of ischemic stroke patients at 11–72 h after admission to the hospital were also reported by Zhou et al. [41]. In addition to blood levels, CSF miR-9 levels are reported to be elevated on D3 after acute ischemic stroke and are associated with a larger infarct size [40]. In contrast to other studies, Liu et al. [44] observed decreased miR-124 and miR-9 levels in the serum of acute ischemic stroke patients (<24 h from stroke onset), and they reported that patients with larger infarct volume had lower miRNA levels.

Interestingly, miR-212 and miR-132, two very closely related miRNAs, yielded different results from each other when their correlation with the structural outcome was investigated. Acute plasma miR-132 levels correlated with larger lesion volume in MRI at all time-points, whereas for miR-212, a correlation was detected only on D21. This suggests that unlike the other investigated miRNAs, increased miR-212 levels in the plasma after TBI may indicate the initiation of pathologic processes becoming evident at later time-points. In addition, miR-212 was the only miRNA in our analysis that did not correlate with the extent of the lesion area at a chronic time-point after TBI. 

To the best of our knowledge, this is the first study assessing the relation between circulating miRNA levels and cortical lesion severity and progression from an acute time-point to a chronic time-point in rats with severe TBI. Taken together, our results indicate that increased levels of neuronally enriched miRNAs in the blood circulation after TBI reflect the extent of cortical injury in the brain. Use of circulating miRNAs as a new tool to evaluate TBI severity and predict disease progression in patients remains to be validated in a clinical setting.

## 4. Materials and Methods

### 4.1. Animals

**Figure 11:**
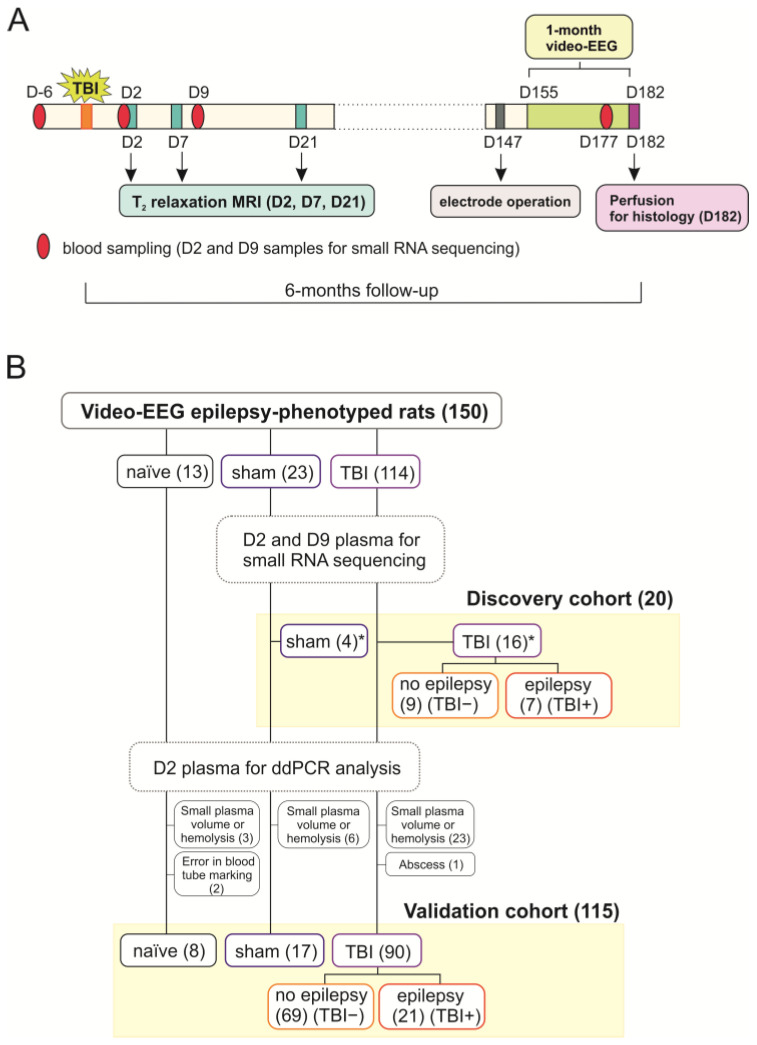
Study design and animal numbers. (**A**) Traumatic brain injury (TBI) was induced in rats by lateral fluid percussion injury. Sham-operated experimental controls underwent the same surgery, including craniotomy without TBI induction. Blood was collected from the tail vein 6 d before (D-6) the injury or sham operation, and then on D2, D9, and D177 after injury. Animals underwent T2 magnetic resonance imaging (MRI) on D2, D7, and D21 to measure cortical lesion volume. To monitor the occurrence of spontaneous seizures (i.e., to diagnose PTE), rats were continuously monitored with vEEG (24/7) for 1 month during the sixth post-injury month. (**B**) A total of 150 animals (13 naïve, 23 sham, 114 TBI) of the EPITARGET cohort were epilepsy-phenotyped using vEEG. Then, D2 and D9 plasma of 20/150 rats, including 4 sham and 16 TBI animals (7 with epilepsy [TBI+], 9 without epilepsy [TBI−]) were used for small RNA sequencing (discovery cohort). The discovery cohort included samples from the first 20 epilepsy-phenotyped rats with acceptable sample quality and sample volume sufficient for small RNA sequencing (see Methods). Validation of miRNA-sequencing data using ddPCR was performed in the plasma of 115 rats [validation cohort; 8 naïve, 17 sham, and 90 TBI animals (21 TBI+, 69 TBI−)]. Abbreviations: D, day; vEEG, video-electroencephalography; *, 2 samples from sham-operated and 5 samples from injured animals (3 TBI−, 2 TBI+) that were used for small RNA sequencing in the discovery cohort were also included in the ddPCR analysis in the validation cohort.

The study design is summarized in Figure 11. The EPITARGET animal cohort included 257 adult male Sprague Dawley rats (Envigo Laboratories S.r.l, Udine, Italy). The mean weight of rats in the EPITARGET cohort was 356 ± 13 g (median 356 g, range 326–419 g) at the time of injury or sham operation. Rats were housed in individual cages in a controlled environment (temperature 22 ± 1 °C, humidity 50–60%, lights on 07:00–19:00) and had ad libitum access to food and water.

The 257 rats were randomized into naïve animals (*n* = 16), sham-operated experimental controls (*n* = 27), and rats with TBI (*n* = 214). From these, a total of 150 rats (13 naïve, 23 sham, 114 TBI [31 rats with epilepsy and 83 rats without epilepsy]) completed the 6-month follow-up, including vEEG monitoring.

The 150 rats included in the plasma miRNA analysis were divided into a discovery cohort and a validation cohort. Samples from the ***discovery cohort*** were used for the small RNA sequencing experiment and contained 4 sham-operated experimental controls and 16 rats with TBI (7 with epilepsy [TBI+] and 9 without epilepsy [TBI−]). The ***validation cohort*** used for digital drop polymerase chain reaction (ddPCR) analysis comprised a total of 115 rats, including 8 naïve, 17 sham, and 90 TBI rats (21 TBI+, 69 TBI−). 

A detailed description of the procedures was reported previously [17,20]. All experiments were approved by the Animal Ethics Committee of the Provincial Government of Southern Finland and performed in accordance with the guidelines of the European Community Council Directives 2010/63/EU.

### 4.2. Induction of TBI by LFPI

TBI was induced by LFPI as described previously [17]. The impact pressure was adjusted to produce severe TBI with an expected acute post-impact mortality of 20–30% within the first 48 h. The mean impact pressure in the EPITARGET cohort was 3.25 ± 0.10 atm (*n* = 212, median 3.3. atm, range 2.5–3.6 atm). Time in apnea and the occurrence and duration of impact-related seizure-like behaviors were monitored and documented using project-tailored common data elements (https://epitarget.eu/, accessed on 14 November 2022).

### 4.3. Blood Collection

Blood was sampled 7 days (D) before injury (baseline), and thereafter on D2 (48 h after impact), D9, and 6 months after injury (day of injury referred as D0). Blood sampling from the tail vein and plasma sample preparation were performed according to [92]. Briefly, rats were anesthetized by isoflurane inhalation (5% induction, 1–2% maintenance). A 24G butterfly needle was used to draw blood from the lateral tail vein into 2 BD Microtainer K_2_ EDTA-tubes (#365975, di-potassium ethylenediaminetetraacetic acid, BD Biosciences, Franklin Lakes, NJ, USA; 500 µL/tube). To obtain plasma, tubes were centrifuged at 1300× *g* for 10 min at 4 °C (5417R Eppendorf, Hamburg, Germany) within 1 h after blood sampling. Plasma aliquots of 50 µL were carefully collected and pipetted into 0.5 mL Protein LoBind tubes (#022431064, Eppendorf, Hamburg, Germany) and stored at −70 °C.

### 4.4. Video-EEG Monitoring

At 5 months after TBI (D147), rats were anesthetized and implanted with 3 skull electrodes. Starting on D154 (1 week after electrode implantation), rats underwent continuous (24 h/day; 7 days/week) vEEG monitoring for 4 weeks to diagnose PTE (for details, see [17]). Seizure occurrence was detected both by visual screening and a seizure detection algorithm. An electroencephalographic seizure was defined as a high-amplitude rhythmic discharge that clearly represented an atypical EEG pattern (i.e., repetitive spikes, spike-and-wave discharges, poly-spike-and-wave, or slow-waves; frequency, and amplitude modulation) lasting >10 s [93]. Rats were defined as having epilepsy if at least 1 unprovoked electrographic seizure was detected. In the EPITARGET cohort of 114 TBI animals, the prevalence of PTE was 25% (29/114).

### 4.5. Analysis of Structural Outcome

#### 4.5.1. Magnetic Resonance Imaging and Lesion Analysis

Rats were imaged on D2, D7, and D21 using quantitative T_2_ magnetic resonance imaging (MRI), as described previously [20]. Imaging voxels within the cortex were classified as normal or abnormal based on their T_2_ values. The range for normal T_2_ was defined as 45 ms ≤ T_2_ ≤ 55 ms, with the lower limit corresponding to the 2.5th percentile and the upper limit corresponding to the 97.5th percentile of all imaging voxels of all sham-operated controls across all time-points. Values below the lower limit or above the upper limit were classified as abnormal. To estimate the cortical lesion volume for each animal, the number of abnormal voxels was counted and multiplied by voxel size.

#### 4.5.2. Histology and Preparation of Unfolded Maps

Details of the histology and preparation of cortical unfolded maps were previously described [35] and are only briefly summarized here. 

***Perfusion***. Deeply anesthetized rats were intracardially perfused with 0.9% NaCl followed by 4% paraformaldehyde in 0.1 M sodium phosphate buffer (PB) after completing the vEEG monitoring (D182). The brain was removed from the skull and fixed in 4% paraformaldehyde for 4 h, cryoprotected in 20% glycerol in 0.02 M potassium phosphate-buffered saline (KPBS, pH 7.4) for 24 h, frozen in dry ice, and stored in −70 °C for further processing. Frozen coronal sections of the brain were cut (25-µm thick, 1-in-12 series) using a sliding microtome. The first series of sections was stored in 10% formalin at room temperature and used for thionin staining. Other series of sections were collected into tissue collection solution (30% ethylene glycol, 25% glycerol in 0.05 M PB) and stored at −20 °C until processed.

***Nissl staining***. The first series of sections was stained with thionin, cleared in xylene, and cover-slipped using Depex^®^ (BDH Chemical, Poole, UK) as a mounting medium.

***Preparation of cortical unfolded maps***. To assess the cortical lesion area and the damage to different cytoarchitectonic cortical areas after TBI, thionin-stained sections were digitized (40×, Hamamatsu Photonics, Hamamatsu, Japan; NanoZoomer-XR, NDP.scan 3.2]. Unfolded cortical maps were then prepared from the digitized histologic sections as described in detail by [94] and by applying in-house software from https://unfoldedmap.org (accessed on 14 November 2022) adapted to the rat brain [95].

### 4.6. Small RNA Sequencing from Plasma

#### 4.6.1. Library Preparation and Sequencing

Sequencing was performed for D2 and D9 plasma samples collected from 4 sham-operated controls and 16 TBI rats (7 with epilepsy [TBI+] and 9 without epilepsy [TBI−]). For each animal, 5 frozen 50-μL aliquots (total 250 μL) were pooled for RNA extraction at each time-point. Any visible hemolysis in the plasma samples was visually inspected by an experienced researcher before pooling the aliquots and further confirmed by measurement of hemoglobin absorbance at 414 nm using a spectrophotometer (NanoDrop 2000, Thermo Fisher Scientific, Wilmington, DE, USA). Samples were considered hemolyzed if the A414 value was ≥0.25 (see [92]).

RNA was extracted from 200 μL plasma using an miRNeasy Mini Kit (#217004, QIAGEN, Hilden, Germany). Small RNA sequencing was conducted by GenomeScan (Leiden, the Netherlands). Small RNA library preparation was performed using the Illumina TruSeq Small RNA Sample Prep Kit (Illumina, San Diego, CA, USA). Briefly, small RNA was isolated from purified RNA by size selection after adapter ligation. The excised product was used for PCR amplification of the resulting product. The quality and yield after sample preparation were measured with a Fragment Analyzer. The size of the resulting products was consistent with the expected size of approximately 150 bp. Single-end sequencing with a read length of 51 nucleotides was performed on the Illumina HiSeq 4000. To increase read depth each sample was sequenced on two flow-cells giving two replicates for each sample.

#### 4.6.2. Quantification of miRNAs and Differential Expression Analysis

Read quality of the raw data was assessed using FastQC v11.8 software produced by the Babraham Institute (Babraham, Cambridgeshire, UK) and the Trimmomatic v0.36 was used to filter low-quality base calls and any adapter contamination [96]. Low-quality leading and trailing bases were removed from each read, and a sliding window trimming using a window of 4 and a phred33 score threshold of 15 was used to assess the quality of the read body. Any reads <17 nucleotides were discarded. After quality control, the replicates for each sample were merged and aligned to the rat reference genome (Rno6) using Bowtie2 with the—very-sensitive-local settings. Using the featureCounts program from the Subread package, the number of reads aligned with the known rat miRNAs in accordance with miRBase22 was calculated [97,98].

Following the primary quantification, differential expression analysis was performed with DESeq2 (v. 1.30.1) in RStudio (v. 1.1.463) using R (v. 4.0.2). D2 samples and D9 samples were analyzed separately. First, the raw read table was filtered to exclude miRNAs with no reads in any of the samples. Next, DESeq2 analysis was run to compare TBI vs. sham, TBI+ vs. sham, TBI− vs. sham, and TBI+ vs. TBI− groups. MicroRNAs with an adjusted *p*-value < 0.05 were considered differentially expressed.

#### 4.6.3. Identification of Expression Pattern Differences with Machine Learning

We applied logistic regression with feature selection, utilizing nested leave-1-out cross-validation to identify miRNAs (“features”) that contributed the most to the group differences between sham and TBI, and between TBI− and TBI+. The logistic regression model was optimized on raw counts from miRNAs with counts ≥1 in at least 80% of the samples to maximize separation between groups in terms of the area under the curve (AUC) of the receiver operating characteristic (ROC) curve. The miRNA counts were standardized to 0 mean and unit variance. In the inner cross-validation loop, miRNAs with 0 variance were filtered and feature selection was performed using recursive feature elimination and filtering by F-score. Model hyperparameters and feature selection configurations were optimized with a grid search over combinations of regularization factor levels, feature selection methods, number of selected features, and choices between L1 (LASSO) and L2 (Ridge) regularization. Feature importance was calculated by averaging the absolute values of logistic regression model covariates over the outer fold of nested cross-validation. The averaged values were normalized to sum to 1 and organized in descending order to identify miRNAs that contributed the most to group separability. Analyses were performed using Python (3.7.0) and the sklearn package (20.2) on Centos 7.

#### 4.6.4. Visualization of Sequencing Data

Shared miRNAs between the experimental groups in small RNA sequencing were visualized using Venn diagrams (https://bioinfogp.cnb.csic.es/tools/venny/, accessed on 6 October 2022).

Normalization of the raw read counts to counts per million (CPM) was performed using the Equation (1):

(1) $$CPM=\frac{read\;count\;per\;gene}{total\;read\;count\;per\;sample}\times 1,000,000$$

Normalized data were visualized using principal component analysis (PCA), Spearman correlation matrices, and heatmaps with a complete linkage clustering method and Spearman correlation as the distance measurement. PCA plots, correlation plots, and heatmaps were prepared using RStudio (v. 1.1.463) by R (v. 4.0.2).

### 4.7. Technical Validation of miRNA-Sequencing Data by RT-qPCR

#### 4.7.1. MiRNA Extraction from Plasma

MicroRNA was extracted from 50-µL plasma samples of 20 rats (4 sham, 16 TBI) using an miRNeasy Mini Kit (#217004, QIAGEN) with a final elution volume of 30 µL. These plasma samples came from the same rats used for sequencing, but the aliquots were different from those used for sequencing.

#### 4.7.2. Reverse Transcription

Reverse transcription (cDNA synthesis) was conducted using a miRCURY LNA RT Kit (#339340, QIAGEN). In each reaction, 2 µL of template miRNA was used. The following temperature cycling protocol was used: incubation for 60 min at 42 °C followed by incubation for 5 min at 95 °C to heat activate the reverse transcriptase, and immediate cooling to 4 °C.

#### 4.7.3. RT-qPCR

Quantitative PCR was conducted using miRCURY LNA SYBR Green PCR Kit (#339345, QIAGEN) and miRCURY LNA miRNA PCR assays (hsa-miR-9-3p, #YP00204620; mmu-miR-434-3p, #YP00205190; hsa-miR-323a-3p, #YP00204278; hsa-miR-136-3p, #YP00205503; hsa-miR-129-5p, #YP00204534; hsa-miR-28-3p, #YP00204119, QIAGEN). We selected miR-28-3p as an endogenous control based on geNorm analysis (https://genorm.cmgg.be/, accessed on 10 September 2019) of the sequencing data. The analysis identified miR-28-3p as the most stable miRNA across the sequencing samples on D2 and D9. 

Template cDNA was diluted 1:30 (miR-9a-3p, miR-434-3p, and miR-28-3p) or 1:10 (miR-136-3p, miR-129-5p, and miR-323-3p) in nuclease-free water. The following PCR cycling conditions were used: 2 min heat activation at 95 °C, 40 cycles of 10 s denaturation at 95 °C, and annealing/extension for 60 s at 56 °C. Quantitative PCR was performed using a LightCycler 96 Instrument (Roche, Basel, Switzerland) and LightCycler 96 v. 1.1 software (Roche). 

Data were normalized to miR-28-3p using the formula 2^−ΔCt^. Results were analyzed by GraphPad Prism 8 software (GraphPad Software, San Diego, CA, USA).

### 4.8. DdPCR Analysis

#### 4.8.1. Plasma Quality Control

For the ddPCR analysis, plasma quality was controlled by measuring hemolysis (absorbance at wavelength 414 nm) by a NanoDrop ND-1000 spectrophotometer (Thermo Fisher Scientific, Waltham, MA, USA). Samples were considered hemolyzed if the A414 value was ≥0.25 [92]. 

First, A414 was measured from one 50 µL plasma aliquot (3rd of the 4 aliquots). If A414 was <0.25, another 3 aliquots (50 µL each) were thawed. The four aliquots were pooled to obtain a total of 200 µL plasma, and A414 was measured again from the pooled plasma sample.

#### 4.8.2. MiRNA Extraction from Plasma

MicroRNA was extracted from the pooled 200 µL plasma samples using an miRNeasy Mini Kit following the protocol from the miRNeasy Serum/Plasma kit. The elution volume was 30 µL. Extracted miRNA samples were stored at −70 °C. The small RNA concentration in each sample was determined using the Qubit microRNA Assay Kit (#Q32880, Thermo Fisher Scientific) with a DeNovix DS-11 FX Fluorometer (DeNovix Inc., Wilmington, DE, USA) from a separate 1 µL sample aliquot.

#### 4.8.3. DdPCR Validation of miRNAs

Total RNA was transcribed to cDNA with the miRCURY LNA RT kit as described in the preceding text. The total reaction volume was 30 µL (6 µL template RNA in each reaction). The cDNA was stored at −20 °C until ddPCR analysis.

For ddPCR analysis, cDNA was diluted 1:10 in nuclease-free water. The master mix for 1 reaction (reaction volume 20 µL) contained 10 µL of 2 X QX200 ddPCR EvaGreen SuperMix (#1864034, BIO-RAD, Hercules, CA, USA), 1.0 µL nuclease-free water, and 1.0 µL miRCURY LNA PCR assay (QIAGEN). Each reaction contained 8 µL of the diluted cDNA template. Droplets were generated using the QX200 AutoDG Droplet Digital PCR System (BIO-RAD) with Automated Droplet Generation Oil for EvaGreen (#1864112, BIO-RAD). Seven target miRNAs (hsa-miR-9-3p, #YP00204620; mmu-miR-434-3p, #YP00205190; hsa-miR-136-3p, #YP00205503; hsa-miR-323a-3p, #YP00204278; hsa-miR-124-3p, #YP00206026; hsa-miR-132-3p, #YP00206035; mmu-miR-212-3p, #YP00206022, QIAGEN) and the endogenous control (miR-28-3p) were all analyzed on the same ddPCR plate, 5 samples per plate. The plate contained 2 replicate wells for each sample. Each plate contained a control sample to monitor possible differences between the ddPCR runs. PCR was conducted using a C1000 Touch™ Thermal Cycler (#1851196, BIO-RAD) with the following program: 95 °C 5 min, 95 °C 30 s, 56 °C 1 min, repeat total 40 cycles, 4 °C 5 min, 90 °C 5 min, 4 °C hold. Droplets were quantified by the QX200 Droplet Reader (#1864003, BIO-RAD). The ddPCR results were analyzed by QuantaSoft software (version 1.7.4.0917). Target miRNA concentrations were normalized to the miR-28-3p concentration (target/reference) to acquire normalized expression values for each sample. Results were visualized by GraphPad Prism (v. 9.0.1, GraphPad software, San Diego, CA, USA).

### 4.9. DdPCR Validation of Downregulated miRNAs

From the list of differentially expressed miRNAs between the TBI and sham groups on D2, we selected 3 downregulated miRNAs for further validation: miR-140-3p, miR-149-5p, and miR-455-5p. These 3 miRNAs were selected as they had a low adjusted p-value, log2FC ≤ −1.00, with a mean CPM ≥ 100 in both the TBI and sham samples.

To save the valuable plasma from the EPITARGET cohort, we used D2 plasma from 5 sham and 5 TBI rats available from another animal cohort in our laboratory for ddPCR validation. In addition, baseline samples (D-7) from 1 sham and 4 TBI rats were used as naïve samples (*n* = 5). The mean weight of these rats was 357 ± 14 g (*n* = 34, median: 356 g, range 334−389 g) at the time of TBI or sham operation. TBI was induced as described above. Mean impact pressure was 3.36 ± 0.09 atm (*n* = 25, median: 3.4 atm, range 3.2−3.5 atm). Plasma was collected on D2 after TBI or sham operation. 

RNA was extracted from 188 µL of plasma using the miRNeasy Mini Kit (30 µL elution volume). The ddPCR analysis was conducted using miRCURY LNA miRNA PCR assays (hsa-miR-140-3p, #YP00204304; hsa-miR-455-5p, #YP00204363; hsa-miR-149-5p, #YP00204321). For ddPCR analysis, cDNA was diluted in nuclease-free water as follows: 1:200 dilution for miR-140-3p analysis, 1:10 dilution for miR-455-5p and miR-149-5p analyses. The ddPCR analysis was conducted similarly as described above.

### 4.10. Glmnet Logistic Regression Analysis

We investigated the optimal combination of analyzed plasma miRNAs as a biomarker panel for predicting epileptogenesis and epilepsy severity by conducting an elastic-net-based analysis using glmnet (https://hastie.su.domains/glmnet_matlab/, accessed on 14 November 2022) [99,100] for MATLAB (R2017a, the MathWorks Inc., Natick, MA, USA). Elastic net uses least absolute shrinkage selector operator (LASSO) and Ridge regularization to reduce overfitting of the models, and we selected equal weighting for the 2 [101]. Nested (externally validated) leave-1-out cross-validation was used to avoid overfitting the regularization parameter [102,103]. Observations were weighted to compensate for class imbalance, and the fitting was performed by minimizing binomial deviance. Normalized expression levels of 7 miRNAs in the plasma on D2 after TBI were used as predictor variables (miR-434, miR-9, miR-136, miR-323, miR-124, miR-212, and miR-132). The predictor variables that had a coefficient of zero in the majority of the outer cross-validation folds after the glmnet fit were excluded [104]. The remaining predictors were used in a standard (non-regularized) logistic regression analysis (MATLAB function “fitglm”). For the TBI+ vs. TBI− analysis, the response variable was epilepsy (yes/no) determined by the vEEG analysis. For the epilepsy severity analyses, the response variable was seizure clusters (yes/no) or at least 3 seizures during the follow-up (yes/no). Cross-validated ROC AUC was computed as a measure of goodness of fit for the logistic regression models using the pooling method [105] ROC AUC and its 95% confidence interval were estimated with Brian Lau’s MatlabAUC codes (https://github.com/brian-lau/MatlabAUC, accessed on 14 November 2022). The confidence interval was estimated as a bias-corrected and accelerated bootstrap using 10,000 samples.

### 4.11. Ingenuity Pathway Analysis

Ingenuity pathway analysis (IPA, QIAGEN, version 76765844) was used to investigate miRNA target genes and their function. Because miR-132-3p and miR-212-3p are closely related and share the same seed sequence, IPA only used miR-132-3p for target prediction in the microRNA Target Filter. The resulting target genes were further analyzed by Expression Analysis in IPA to reveal the canonical pathways associated with the target genes. “Experimentally observed” and “High (predicted)” confidence settings were applied in both the microRNA Target Filter and Expression Analysis.

### 4.12. Statistics

Differential miRNA expression analysis was performed with DESeq2. Differential expression was considered at a level of FDR < 0.05. Other statistical analyses were performed using GraphPad Prism 9 and RStudio (v. 1.1.463) by R (v. 4.0.2). Comparisons of 3 or more groups were performed using the Kruskal–Wallis test, followed by *post hoc* analysis using the Mann–Whitney U test. Correlations were analyzed by the Spearman correlation test (*ρ*). The ROC analysis was performed for each ddPCR-validated miRNA to assess its sensitivity and specificity in differentiating the animal groups. Statistical significance of the AUC was assessed by Mann–Whitney U test. The optimal cut-off in ROC analysis was determined using the cutpointr package (v. 1.1.1) in R by maximizing the sum of sensitivity and specificity. Results are presented as mean±standard deviation. A *p*-value < 0.05 was considered statistically significant.

## 5. Conclusions

We validated the upregulation of seven brain-enriched miRNAs in the plasma on D2 after LFPI-induced TBI in the EPITARGET study cohort. The plasma miRNA expression profile on D2 after TBI did not predict the subsequent development of PTE or the PTE severity. Our data did, however, reveal that acute post-TBI plasma miRNA levels predicted the severity of the cortical pathology at acute, subacute, and chronic time-points. Moreover, we found that six miRNAs, including miR-434-3p, miR-9a-3p, miR-136-3p, miR-323-3p, miR-212-3p, and miR-132-3p, differentiated naïve rats from sham-operated rats, demonstrating the capability to detect mild brain injury caused by the craniotomy.

## Data Availability

The small RNA sequencing data has been deposited to the Gene Expression Omnibus (GEO) under accession No. GSE222801. Further data are available on request from the corresponding author.

## Supplementary Materials

The following supporting information can be downloaded at https://www.mdpi.com/article/10.3390/ijms24032823/s1.
