# Supplementary material for: Discovery and Validation of Circulating microRNAs as Biomarkers for Epileptogenesis after Experimental Traumatic Brain Injury–The EPITARGET Cohort
Source: Int J Mol Sci. 2023 Feb 1;24(3):2823. doi: 10.3390/ijms24032823 (PMC9918096; doi:10.3390/ijms24032823)
Supplement: Supplementary file 1 [file ijms-24-02823-s001.zip › ijms-2156089-supplementary.pdf]

Supplementary Data

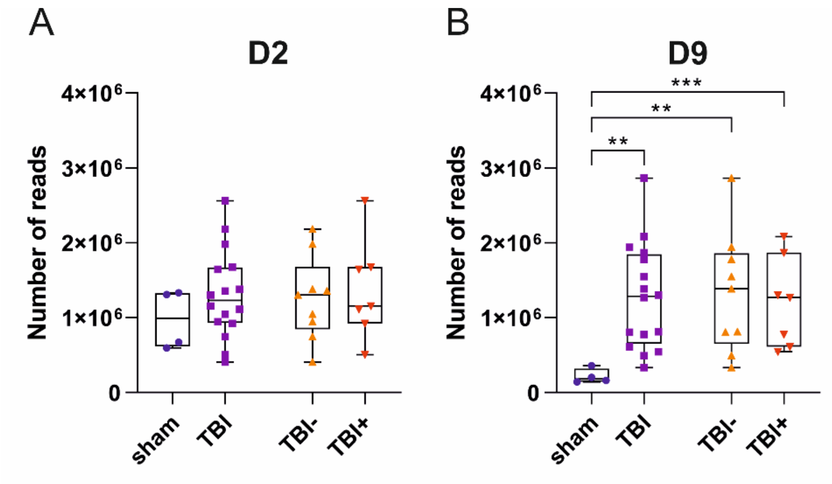

Supplementary Figure S1. Number of reads mapping to miRNAs in small RNA sequencing. (A) In D2 samples, the number of reads mapping to miRNAs did not differ between the groups (4 sham, 16 TBI [9 TBI-, 7 TBI+ rats]). **(B)** In D9 samples, sham-operated controls had fewer mapped reads than the TBI rats. Abbreviations: D, day; TBI, traumatic brain injury; TBI+, TBI rats with epilepsy; TBI-, TBI rats without epilepsy. Statistical significance: Kruskal-Wallis test followed by *post hoc* analysis with Mann-Whitney U test: **, p<0.01; ***, p<0.001 (Mann-Whitney U test).

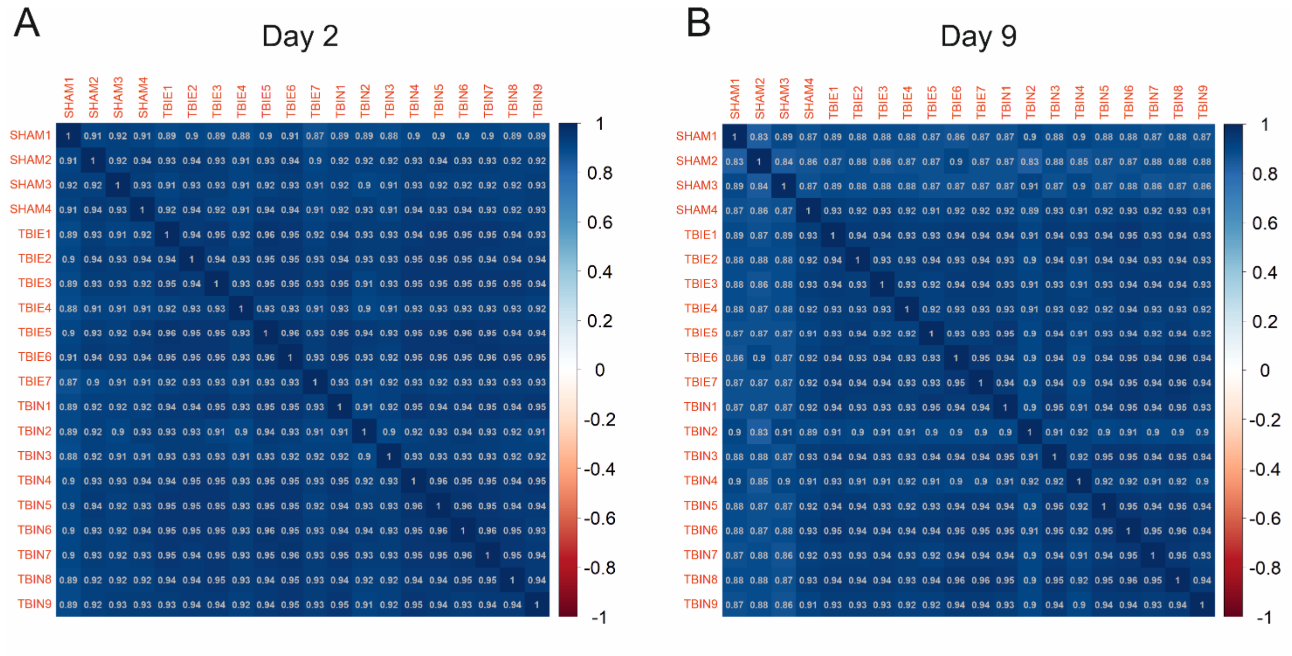

Supplementary Figure S2. Spearman correlation plots for plasma microRNAs (miRNAs) in TBI and sham groups. High positive correlation coefficients were observed between all samples on both **(A)** D2 and **(B)** D9, indicating similar overall miRNA expression profiles between TBI and sham groups. Abbreviations: D, day; TBI, traumatic brain injury.

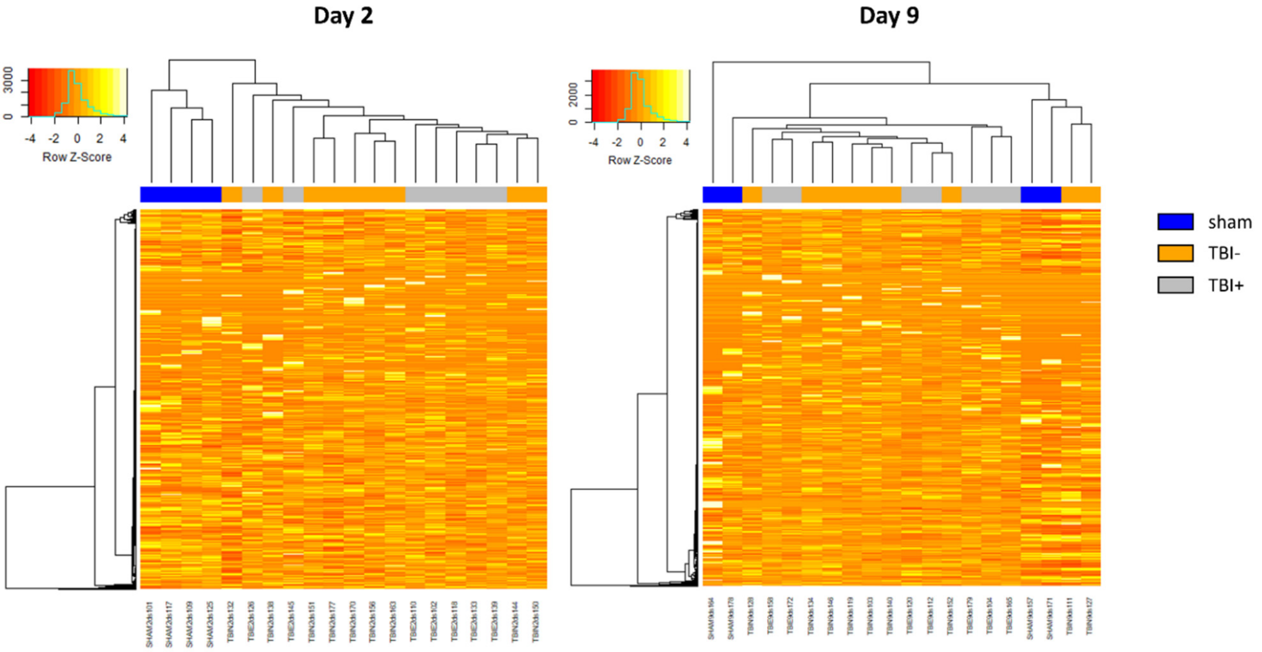

(A) (B)

Supplementary Figure S3. Heatmaps of plasma miRNA expression in sham and TBI rats. (A) Expression of miRNAs in plasma on D2 separated sham-operated rats (n=4) from the TBI group (n=16). **(B)** The expression of miRNAs in plasma on D9 did not separate sham-operated controls from the TBI group. No clear separation of rats with (TBI+, 7/16) or without epilepsy (TBI-, 9/16) was detected on either day. Abbreviations: TBI, traumatic brain injury.

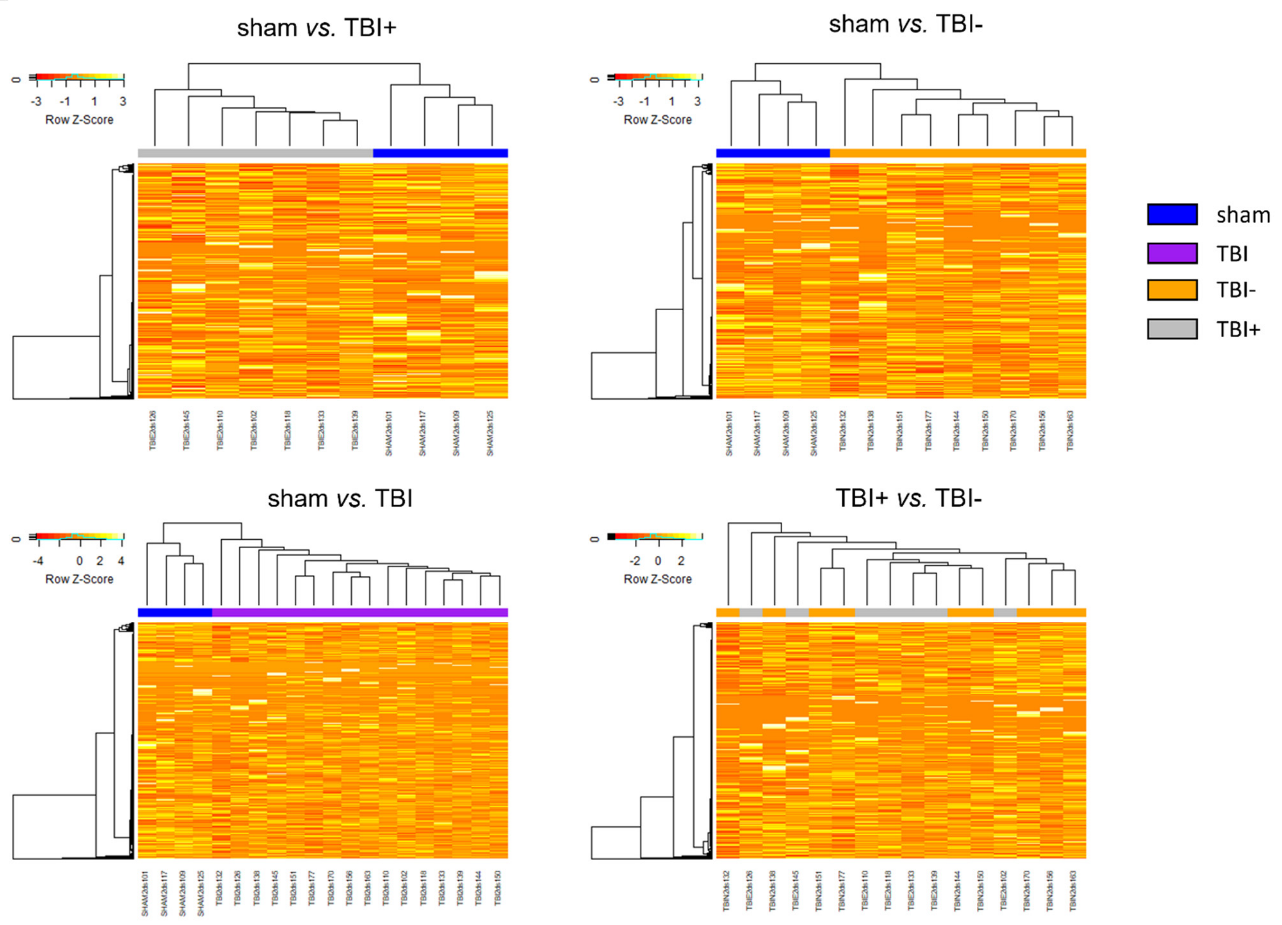

Supplementary Figure S4. Pairwise comparison heatmaps of plasma miRNA expression on D2. Expression profile of plasma miRNAs on D2 separated sham-operated rats (n=4) from rats with (TBI+, n=7) or without (TBI-, n=9) epilepsy, and from all TBI rats combined (n=16). The expression profile of plasma miRNAs did not separate animals with (TBI+) or without (TBI-) epilepsy. Abbreviations: TBI, traumatic brain injury.

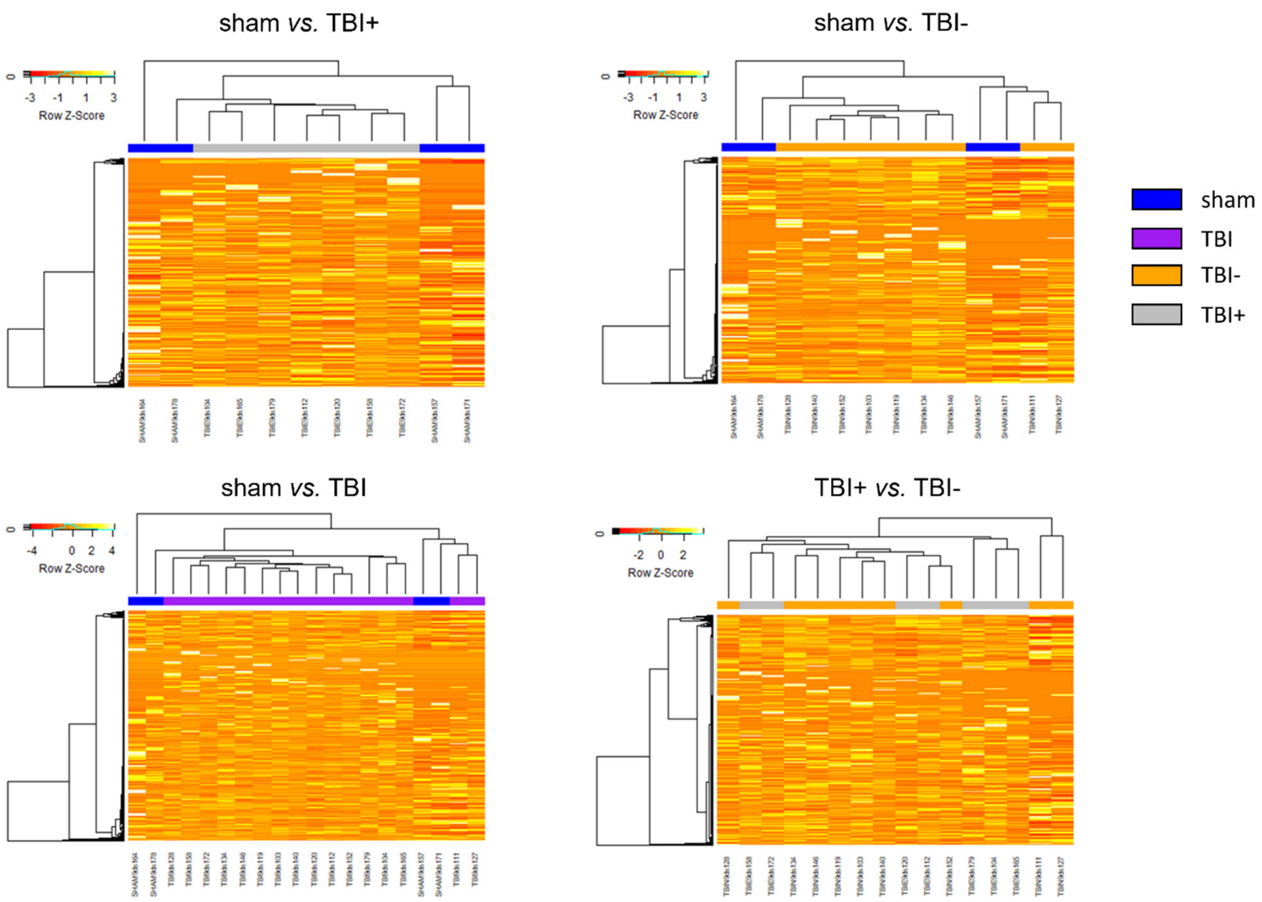

Supplementary Figure S5. Pairwise comparison heatmaps of plasma miRNA expression D9. Expression profile of plasma miRNAs on D9 did not separate sham-operated rats (n=4) from rats with (TBI+, n=7) or without (TBI-, n=9) epilepsy, or from all TBI rats combined (n=16). The expression profile of plasma miRNAs did not separate TBI+ from TBI-. Abbreviations: TBI, traumatic brain injury.

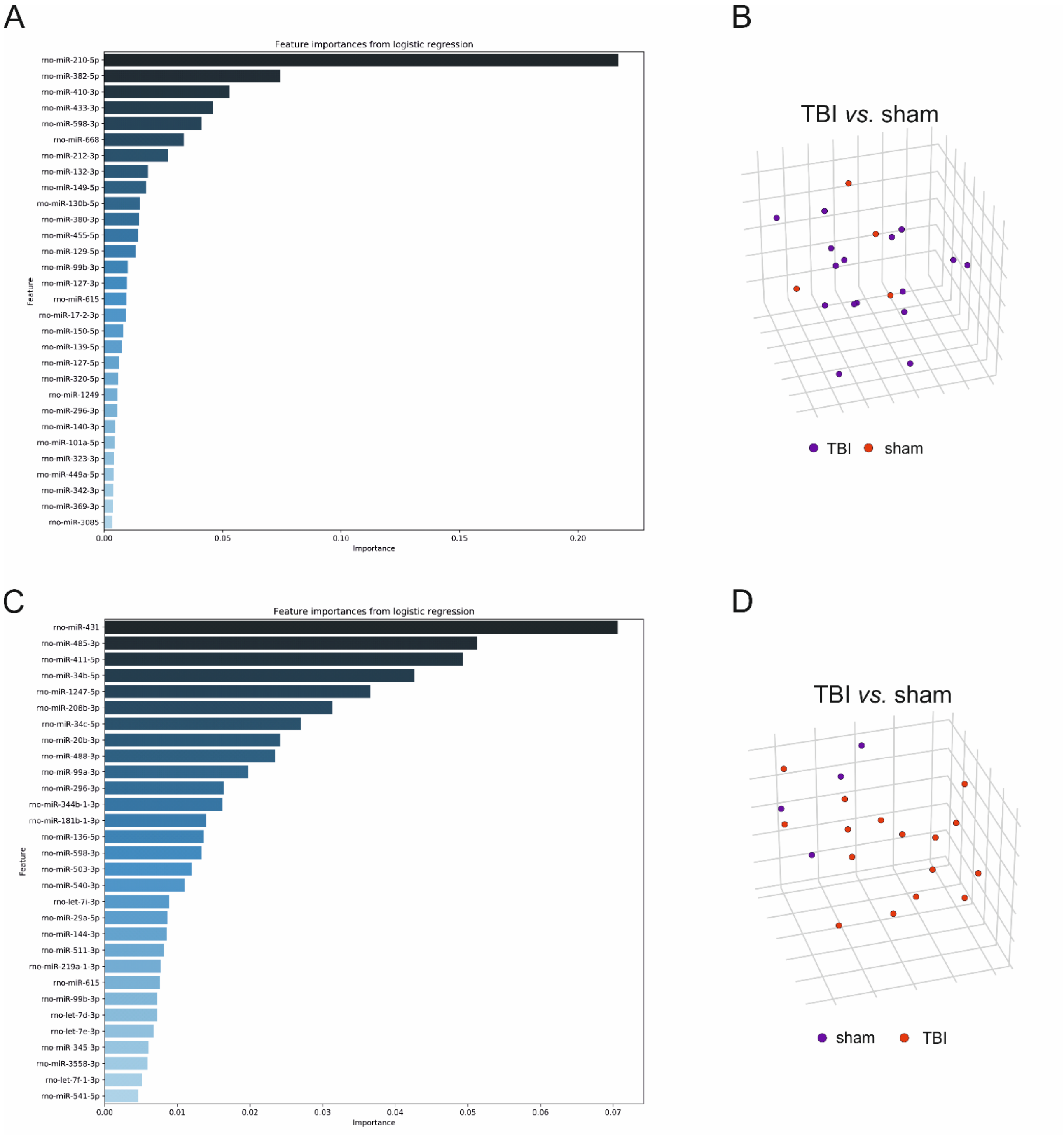

Supplementary Figure S6. *Machine learning analysis of plasma miRNAs to differentiate sham and TBI groups.* **(A)** Feature importance from logistic regression models optimized for sham vs TBI classification on D2. The feature importance denotes the absolute values of model coefficients averaged over cross-validation (CV) folds and normalized to sum to 1. **(B)** t-SNE reduction of raw read counts of miRNAs on D2. The included miRNAs had a count ≥1 in at least 80% of samples. **(C)** Feature importance from logistic regression models optimized for sham vs TBI classification on D9. **(D)** t-SNE reduction of raw read counts of miRNAs on D9.

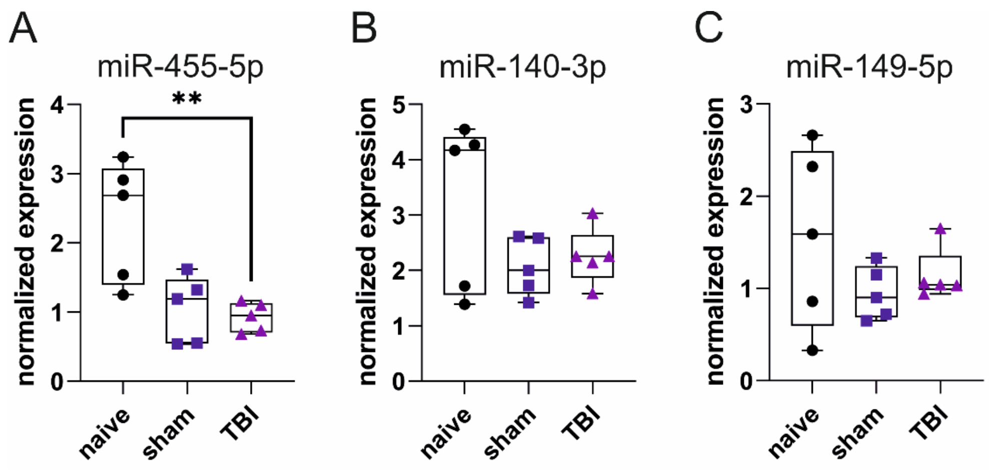

Supplementary Figure S7. ***ddPCR analysis of downregulated miRNAs.*** **(A)** Downregulated miRNAs were measured in D2 plasma samples of 5 naïve (baseline), 5 sham-operated, and 5 TBI rats. In TBI animals, normalized plasma miR-455-5p levels were decreased to 40% of that in naïve rats (Mann-Whitney U test p<0.01). No differences were detected in **(B)** miR-140-3p or **(C)** miR-149-5p levels between groups (Kruskal-Wallis test, p>0.05). Abbreviations: TBI, traumatic brain injury.

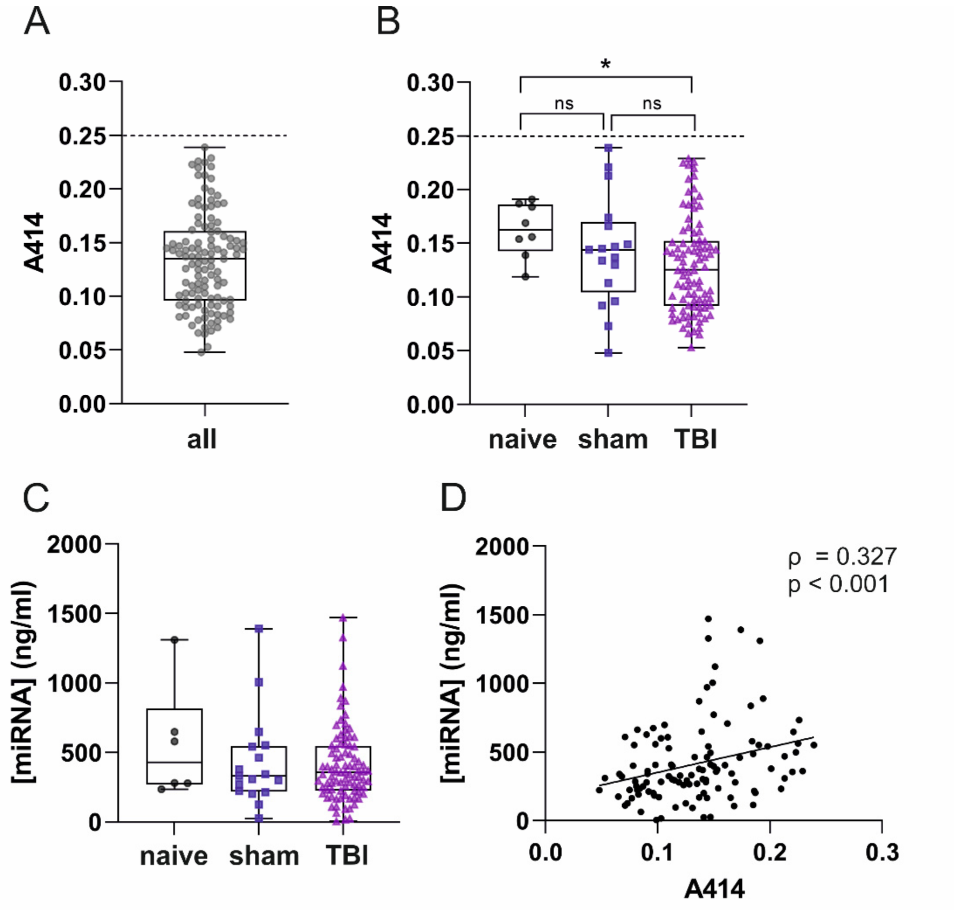

Supplementary Figure S8. Plasma hemolysis and total miRNA concentration. (A) Hemolysis in pooled plasma samples from the rats included in the EPITARGET validation cohort (n=115) measured by NanoDrop (absorbance at 414 nm, A414). Dashed line shows the cut-off value (0.25) for the hemolyzed samples. All samples had absorbance below the cut-off. **(B)** Hemolysis in the EPITARGET validation cohort (8 naïve, 17 sham, 90 TBI). Samples in the TBI group had slightly lower absorbance values than those in the naïve group (Mann-Whitney *U* test, $p<0.05$). **(C)** miRNA concentrations measured by the Qubit microRNA Assay kit did not differ between the experimental groups (Kruskal-Wallis test, $p>0.05$). Results are from 6 naïve, 17 sham, and 88 TBI samples (2 naïve and 2 TBI samples had miRNA concentration below the Qubit detection limit). **(D)** A414 values (hemolysis) showed a weak positive correlation with the total miRNA concentration (n=111, Spearman r=0.327, $p<0.001$).

A

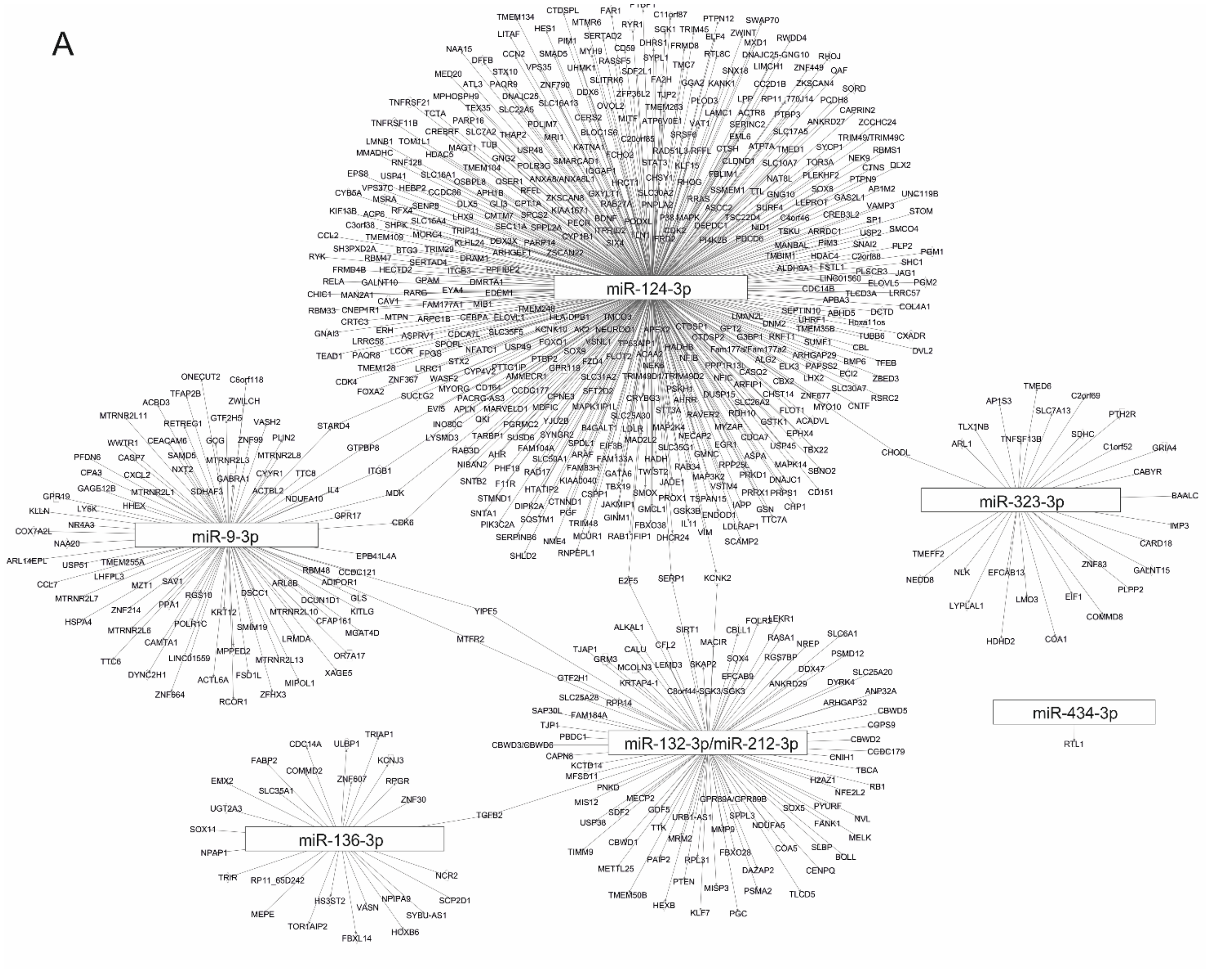

B

Top canonical pathways of miRNA targets

miR-124-3p	p-value	Overlap
Molecular Mechanisms of Cancer	4.62E-08	6.3% (28/446)
Aryl Hydrocarbon Receptor Signaling	2.45E-06	8.8% (14/159)
MSP-RON Signaling in Cancer Cells Pathway	3.10E-06	9.3% (13/140)
Apelin Endothelial Signaling Pathway	3.35E-06	9.2% (13/141)
Senescence Pathway	5.75E-06	6.4% (19/299)

miR-132-3p/miR-212-3p	p-value	Overlap
Kinetochore Metphase Signaling Pathway	6.91E-04	3.6% (4/111)
Senescence Pathway	7.31E-04	2.0% (6/299)
Pancreatic Adenocarcinoma Signaling	1.11E-03	3.2% (4/126)
Adipogenesis Pathway	1.43E-03	3.0% (4/135)
Cell Cycle: G1/S Checkpoint Regulation	1.89E-03	4.4% (3/68)

miR-9-3p	p-value	Overlap
Hematopoiesis from Multipotent Stem Cells	7.64E-04	16.7% (2/12)
Glutamine Degredation I	6.91E-03	50% (1/2)
Huntington's Disease Signaling	1.69E-02	1.4% (4/283)
Granulocyte Adheasion and Diapedesis	2.80E-02	1.6% (3/189)
TREM1 Signaling	2.93E-02	2.6% (2/77)

miR-136-3p	p-value	Overlap
WNT/ß-catenin Signaling	1.36E-02	1.1% (2/174)
Natural Killer Cell Signaling	1.74E-02	1.0% (2/198)
Apelin Cardiac Fibroblast Signaling Pathway	2.33E-02	4.3% (1/23)
LPS/IL-1 Mediated Inhibition of RXR Function	2.77E-02	0.8% (2/254)
Airway Inflammation in Asthma	3.33E-02	3.0% (1/33)

miR-323-3p	p-value	Overlap
Macrophage Classical Activation Signaling Pathway	2.14E-02	1.1% (2/189)
TCA Cycle II (Eukaryotic)	2.72E-02	4.3% (1/23)
B Cell Activating Factor Signaling	5.02E-02	2.3% (1/43)
Role of Macrophages, Fibroblasts and Endothelial Cells in Rheumatoid Arthritis	5.96E-02	0.6% (2/332)
Triacylglycerol Biosynthesis	6.04E-02	1.9% (1/52)

Supplementary Figure S9. Ingenuity Pathway Analysis (IPA) of miRNA target genes. (A) Qiagen microRNA Target Filter in IPA found 1 target gene to miR-434-3p, 89 to miR-9-3p, 27 to miR-136-3p, 29 to miR-323-3p, 460 to miR-124-3p, and 91 to miR-132-3p/miR-212-3p. The investigated miRNAs shared only a few common target genes. **(B)** Pathway analysis of miRNA target genes revealed different top 5 canonical pathways for miR-124-3p, miR-9-3p, miR-323-3p, miR-132-3p/miR-212-3p, and miR-136-3p. No canonical pathways were found for miR-434-3p as miR-434-3p had only 1 target gene in the analysis. Canonical pathways are considered significantly enriched if p≤0.05.

Supplementary Table S1. Differentially expressed miRNAs on D2 after TBI (TBI *vs.* sham animals).

miRNA	baseMean	log2FC	lfcSE	stat	pvalue	padj
**Upregulated**						
rno-miR-124-3p	27.89	7.44	1.09	6.85	7.17E-12	1.20E-09
rno-miR-129-5p	73.98	2.57	0.39	6.56	5.44E-11	6.06E-09
rno-miR-124-5p	17.34	6.75	1.08	6.22	4.91E-10	4.10E-08
rno-miR-127-3p	521.36	1.29	0.22	5.74	9.48E-09	5.28E-07
rno-miR-410-3p	94.93	1.48	0.28	5.30	1.19E-07	5.66E-06
rno-miR-132-3p	66.76	1.37	0.26	5.21	1.93E-07	7.58E-06
rno-miR-129-1-3p	26.62	2.63	0.51	5.20	2.04E-07	7.58E-06
rno-miR-129-2-3p	26.70	2.64	0.51	5.13	2.93E-07	9.79E-06
rno-miR-212-3p	39.00	1.30	0.27	4.87	1.13E-06	3.13E-05
rno-miR-136-3p	77.80	1.17	0.24	4.81	1.49E-06	3.82E-05
rno-miR-668	29.99	2.01	0.42	4.79	1.66E-06	3.96E-05
rno-miR-341	131.13	1.14	0.27	4.24	2.25E-05	5.00E-04
rno-miR-433-3p	16.76	1.55	0.39	3.97	7.16E-05	1.49E-03
rno-miR-323-3p	34.75	1.56	0.40	3.88	1.05E-04	2.06E-03
rno-miR-182	2291.18	1.35	0.36	3.77	1.65E-04	3.07E-03
rno-miR-598-3p	16.85	2.03	0.56	3.64	2.75E-04	4.18E-03
rno-miR-380-3p	5.46	3.12	0.94	3.31	9.34E-04	1.23E-02
rno-miR-139-5p	140.18	0.89	0.27	3.28	1.04E-03	1.25E-02
rno-miR-3085	13.08	1.86	0.57	3.28	1.04E-03	1.25E-02
rno-miR-300-3p	68.88	0.94	0.29	3.22	1.28E-03	1.41E-02
rno-miR-99b-3p	13.37	1.21	0.38	3.22	1.30E-03	1.41E-02
rno-miR-382-5p	6.12	2.03	0.64	3.18	1.46E-03	1.52E-02
rno-miR-376b-3p	5.64	2.36	0.78	3.02	2.56E-03	2.44E-02
rno-miR-10a-5p	83022.55	0.58	0.19	3.01	2.65E-03	2.46E-02
rno-miR-10b-5p	83006.47	0.58	0.19	2.99	2.79E-03	2.52E-02
rno-miR-127-5p	12.65	1.41	0.48	2.94	3.31E-03	2.88E-02
rno-miR-1224	8.55	1.59	0.56	2.82	4.86E-03	3.78E-02
rno-miR-153-3p	12.02	1.80	0.65	2.79	5.35E-03	3.97E-02
**Downregulated**						
rno-miR-140-3p	8117.45	-1.05	0.14	-7.52	5.45E-14	1.82E-11
rno-miR-149-5p	356.04	-1.27	0.22	-5.79	7.00E-09	4.68E-07
rno-miR-455-5p	162.61	-1.46	0.30	-4.90	9.69E-07	2.94E-05
rno-miR-342-3p	702.64	-0.89	0.24	-3.72	1.98E-04	3.48E-03
rno-let-7d-3p	2741.24	-0.88	0.24	-3.70	2.14E-04	3.57E-03
rno-miR-222-3p	281.37	-0.67	0.18	-3.66	2.50E-04	3.98E-03
rno-miR-140-5p	47.11	-1.21	0.34	-3.52	4.24E-04	6.16E-03
rno-miR-130b-5p	29.26	-1.09	0.33	-3.30	9.55E-04	1.23E-02
rno-miR-351-5p	1694.50	-0.73	0.22	-3.32	8.93E-04	1.23E-02
rno-miR-17-2-3p	19.46	-1.04	0.32	-3.21	1.31E-03	1.41E-02
rno-miR-18a-3p	65.26	-0.85	0.28	-3.07	2.17E-03	2.20E-02
rno-miR-1249	53.94	-1.16	0.38	-3.05	2.27E-03	2.23E-02
rno-miR-351-3p	9.99	-1.50	0.51	-2.92	3.45E-03	2.88E-02

rno-miR-542-3p	126.73	-0.69	0.23	-2.93	3.36E-03	2.88E-02
rno-miR-147	91.48	-0.62	0.21	-2.87	4.06E-03	3.31E-02
rno-miR-150-5p	19579.93	-0.81	0.29	-2.83	4.68E-03	3.72E-02
rno-miR-532-5p	240.70	-0.43	0.15	-2.81	4.98E-03	3.78E-02

Abbreviations: baseMean, the average of the normalized count values; log2FC, log2 fold change; lfcSE, standard error estimate for the log2 fold change; stat, the value of the test statistic for the gene or transcript; pvalue, p-value of the test for the gene or transcript; padj; adjusted p-value for multiple testing for the gene or transcript.

Supplementary Table S2. Differentially expressed miRNAs on D2 after TBI (TBI- *vs.* sham animals).

miRNA	baseMean	log2FC	lfcSE	stat	pvalue	padj
**Upregulated**						
rno-miR-124-3p	27.89	7.43	1.10	6.75	1.53E-11	4.04E-09
rno-miR-124-5p	17.34	6.76	1.10	6.14	8.25E-10	1.16E-07
rno-miR-129-5p	73.98	2.43	0.42	5.82	5.80E-09	6.11E-07
rno-miR-212-3p	39.00	1.48	0.26	5.61	2.01E-08	1.69E-06
rno-miR-132-3p	66.76	1.47	0.28	5.29	1.20E-07	7.24E-06
rno-miR-127-3p	521.36	1.28	0.25	5.21	1.86E-07	9.78E-06
rno-miR-129-1-3p	26.62	2.49	0.54	4.63	3.59E-06	1.37E-04
rno-miR-129-2-3p	26.70	2.50	0.55	4.58	4.74E-06	1.66E-04
rno-miR-410-3p	94.93	1.33	0.29	4.55	5.36E-06	1.73E-04
rno-miR-668	29.99	2.00	0.45	4.40	1.09E-05	3.05E-04
rno-miR-136-3p	77.80	1.14	0.26	4.31	1.60E-05	3.97E-04
rno-miR-433-3p	16.76	1.65	0.41	4.05	5.16E-05	1.14E-03
rno-miR-139-5p	140.18	1.05	0.28	3.77	1.61E-04	3.23E-03
rno-miR-341	131.13	1.11	0.29	3.76	1.73E-04	3.32E-03
rno-miR-598-3p	16.85	2.17	0.60	3.64	2.71E-04	4.97E-03
rno-miR-323-3p	34.75	1.51	0.44	3.45	5.64E-04	9.50E-03
rno-miR-382-5p	6.12	2.13	0.66	3.24	1.21E-03	1.70E-02
rno-miR-182	2291.18	1.18	0.38	3.08	2.05E-03	2.50E-02
rno-miR-3085	13.08	1.89	0.61	3.09	1.98E-03	2.50E-02
rno-miR-380-3p	5.46	3.00	0.97	3.08	2.08E-03	2.50E-02
rno-miR-300-3p	68.88	0.91	0.32	2.88	3.96E-03	4.51E-02
**Downregulated**						
rno-miR-140-3p	8117.45	-1.00	0.15	-6.71	1.92E-11	4.04E-09
rno-miR-149-5p	356.04	-1.28	0.24	-5.31	1.08E-07	7.24E-06
rno-let-7d-3p	2741.24	-1.13	0.22	-5.12	3.00E-07	1.40E-05
rno-miR-18a-3p	65.26	-1.19	0.25	-4.74	2.17E-06	9.13E-05
rno-miR-1249	53.94	-1.62	0.36	-4.49	7.20E-06	2.17E-04
rno-miR-455-5p	162.61	-1.42	0.33	-4.35	1.33E-05	3.51E-04
rno-miR-222-3p	281.37	-0.80	0.19	-4.27	2.00E-05	4.67E-04
rno-miR-140-5p	47.11	-1.40	0.37	-3.79	1.51E-04	3.17E-03
rno-miR-342-3p	702.64	-0.95	0.26	-3.62	2.89E-04	5.07E-03
rno-miR-210-5p	2.98	-2.32	0.68	-3.43	6.06E-04	9.82E-03
rno-miR-542-3p	126.73	-0.83	0.24	-3.40	6.70E-04	1.04E-02
rno-miR-130b-5p	29.26	-1.21	0.36	-3.33	8.81E-04	1.32E-02
rno-miR-351-5p	1694.50	-0.79	0.24	-3.31	9.28E-04	1.35E-02
rno-miR-17-2-3p	19.46	-1.16	0.36	-3.20	1.37E-03	1.86E-02
rno-miR-24-2-5p	355.54	-0.60	0.19	-3.17	1.51E-03	1.99E-02
rno-miR-351-3p	9.99	-1.72	0.57	-3.02	2.51E-03	2.94E-02

Abbreviations: baseMean, the average of the normalized count values; log2FC, log2 fold change; lfcSE, standard error estimate for the log2 fold change; stat, the value of the test statistic for the gene or transcript; pvalue, p-value of the test for the gene or transcript; padj; adjusted p-value for multiple testing for the gene or transcript.

Supplementary Table S3. Differentially expressed miRNAs on D2 after TBI (TBI+ *vs.* sham animals).

miRNA	baseMean	log2FC	lfcSE	stat	pvalue	padj
**Upregulated**						
rno-miR-124-3p	27.89	7.46	1.11	6.73	1.74E-11	3.38E-09
rno-miR-129-5p	73.98	2.74	0.43	6.37	1.88E-10	2.43E-08
rno-miR-124-5p	17.34	6.73	1.11	6.07	1.27E-09	1.24E-07
rno-miR-410-3p	94.93	1.65	0.30	5.47	4.46E-08	3.47E-06
rno-miR-127-3p	521.36	1.30	0.26	5.07	3.88E-07	2.16E-05
rno-miR-129-1-3p	26.62	2.79	0.55	5.08	3.80E-07	2.16E-05
rno-miR-129-2-3p	26.70	2.79	0.56	4.99	6.19E-07	2.68E-05
rno-miR-136-3p	77.80	1.21	0.27	4.44	8.87E-06	3.45E-04
rno-miR-668	29.99	2.04	0.47	4.36	1.32E-05	4.27E-04
rno-miR-132-3p	66.76	1.24	0.29	4.32	1.59E-05	4.76E-04
rno-miR-341	131.13	1.19	0.31	3.91	9.36E-05	2.60E-03
rno-miR-182	2291.18	1.54	0.40	3.85	1.19E-04	3.09E-03
rno-miR-212-3p	39.00	1.05	0.27	3.81	1.38E-04	3.36E-03
rno-miR-323-3p	34.75	1.62	0.45	3.58	3.42E-04	7.82E-03
rno-miR-433-3p	16.76	1.41	0.42	3.35	8.05E-04	1.67E-02
rno-miR-99b-3p	13.37	1.34	0.40	3.31	9.31E-04	1.67E-02
rno-miR-380-3p	5.46	3.26	0.98	3.32	8.96E-04	1.67E-02
rno-miR-376b-3p	5.64	2.54	0.83	3.08	2.10E-03	3.40E-02
rno-miR-369-3p	16.66	1.43	0.47	3.02	2.49E-03	3.73E-02
rno-miR-128-3p	7708.84	0.72	0.24	2.97	2.98E-03	4.14E-02
rno-miR-598-3p	16.85	1.83	0.62	2.97	2.96E-03	4.14E-02
rno-miR-300-3p	68.88	0.97	0.33	2.94	3.25E-03	4.36E-02
rno-miR-153-3p	12.02	2.06	0.70	2.92	3.46E-03	4.49E-02
rno-miR-3085	13.08	1.82	0.63	2.88	3.92E-03	4.73E-02
rno-miR-96-5p	6.43	2.25	0.79	2.87	4.14E-03	4.73E-02
rno-miR-137-3p	2.13	3.52	1.22	2.89	3.87E-03	4.73E-02
rno-miR-1298	2.71	4.27	1.48	2.87	4.06E-03	4.73E-02
**Downregulated**						
rno-miR-140-3p	8117.45	-1.12	0.16	-7.25	4.22E-13	1.64E-10
rno-miR-149-5p	356.04	-1.26	0.25	-5.01	5.39E-07	2.62E-05
rno-miR-455-5p	162.61	-1.50	0.34	-4.39	1.12E-05	3.96E-04
rno-miR-210-5p	2.98	-2.38	0.71	-3.35	8.16E-04	1.67E-02
rno-miR-147	91.48	-0.78	0.24	-3.31	9.47E-04	1.67E-02
rno-miR-155-5p	154.62	-1.15	0.37	-3.08	2.08E-03	3.40E-02
rno-miR-342-3p	702.64	-0.83	0.27	-3.03	2.42E-03	3.73E-02

Abbreviations: baseMean, the average of the normalized count values; log2FC, log2 fold change; lfcSE, standard error estimate for the log2 fold change; stat, the value of the test statistic for the gene or transcript; pvalue, p-value of the test for the gene or transcript; padj; adjusted p-value for multiple testing for the gene or transcript.

Supplementary Table S4. Differentially expressed miRNAs on D9 after TBI (TBI *vs.* sham animals).

miRNA	baseMean	log2FC	lfcSE	stat	pvalue	padj
**Upregulated**						
rno-let-7a-1-3p	52.03	1.42	0.38	3.78	1.56E-04	9.73E-03
rno-let-7c-2-3p	52.41	1.36	0.37	3.67	2.44E-04	1.15E-02
rno-miR-222-3p	192.56	1.18	0.35	3.40	6.67E-04	1.79E-02
rno-miR-340-3p	8.15	3.82	1.11	3.44	5.91E-04	1.79E-02
rno-miR-128-3p	4179.78	1.18	0.37	3.21	1.34E-03	3.13E-02
rno-miR-301b-3p	796.03	1.14	0.37	3.09	1.98E-03	4.37E-02
**Downregulated**						
rno-miR-7a-5p	17.61	-5.42	1.09	-4.97	6.77E-07	2.54E-04
rno-miR-140-3p	5872.00	-1.09	0.25	-4.41	1.01E-05	1.90E-03
rno-miR-7b	7.77	-6.54	1.53	-4.27	1.95E-05	2.44E-03
rno-miR-25-5p	19.30	-2.11	0.53	-3.99	6.48E-05	6.08E-03
rno-miR-149-5p	155.94	-1.16	0.30	-3.94	8.19E-05	6.14E-03
rno-miR-466c-5p	10.44	-1.75	0.47	-3.71	2.08E-04	1.11E-02
rno-miR-674-5p	9.79	-1.90	0.52	-3.63	2.86E-04	1.18E-02
rno-miR-3577	11.16	-1.43	0.40	-3.60	3.15E-04	1.18E-02
rno-miR-34a-5p	16.13	-1.37	0.40	-3.44	5.84E-04	1.79E-02
rno-miR-150-3p	158.70	-1.31	0.39	-3.41	6.56E-04	1.79E-02
rno-miR-351-5p	1098.20	-1.19	0.37	-3.23	1.22E-03	3.06E-02

Abbreviations: baseMean, the average of the normalized count values; log2FC, log2 fold change; lfcSE, standard error estimate for the log2 fold change; stat, the value of the test statistic for the gene or transcript; pvalue, p-value of the test for the gene or transcript; padj; adjusted p-value for multiple testing for the gene or transcript.

Supplementary Table S5. Differentially expressed miRNAs on D9 after TBI (TBI- *vs.* sham animals).

miRNA	baseMean	log2FC	lfcSE	stat	pvalue	padj
**Upregulated**						
rno-let-7a-1-3p	52.03	1.44	0.40	3.63	2.86E-04	1.20E-02
rno-miR-340-3p	8.15	4.04	1.13	3.58	3.45E-04	1.28E-02
rno-let-7c-2-3p	52.41	1.38	0.39	3.51	4.47E-04	1.49E-02
rno-miR-222-3p	192.56	1.25	0.37	3.36	7.67E-04	1.98E-02
rno-let-7d-3p	1807.55	1.05	0.32	3.28	1.02E-03	2.16E-02
rno-miR-128-3p	4179.78	1.22	0.40	3.01	2.60E-03	4.48E-02
rno-miR-98-3p	11.04	3.02	1.01	3.00	2.68E-03	4.48E-02
rno-let-7f-5p	10537.36	1.09	0.37	2.95	3.20E-03	4.87E-02
**Downregulated**						
rno-miR-7a-5p	17.61	-5.11	1.19	-4.29	1.77E-05	5.92E-03
rno-miR-25-5p	19.30	-2.33	0.57	-4.09	4.31E-05	7.21E-03
rno-miR-7b	7.77	-6.62	1.76	-3.76	1.73E-04	8.82E-03
rno-miR-466c-5p	10.44	-2.01	0.51	-3.91	9.11E-05	8.82E-03
rno-miR-3577	11.16	-1.59	0.43	-3.74	1.84E-04	8.82E-03
rno-miR-150-3p	158.70	-1.55	0.40	-3.83	1.30E-04	8.82E-03
rno-miR-140-3p	5872.00	-1.01	0.27	-3.76	1.67E-04	8.82E-03
rno-miR-146b-5p	510.28	-1.52	0.44	-3.46	5.34E-04	1.49E-02
rno-miR-149-5p	155.94	-1.13	0.32	-3.48	5.09E-04	1.49E-02
rno-miR-194-5p	145.31	-1.75	0.53	-3.30	9.54E-04	2.16E-02
rno-miR-34a-5p	16.13	-1.43	0.43	-3.28	1.03E-03	2.16E-02
rno-miR-1247-3p	4.80	-3.53	1.09	-3.24	1.21E-03	2.39E-02
rno-miR-3068-5p	3.84	-6.80	2.18	-3.11	1.86E-03	3.46E-02
rno-miR-674-5p	9.79	-1.67	0.57	-2.96	3.09E-03	4.87E-02

Abbreviations: baseMean, the average of the normalized count values; log2FC, log2 fold change; lfcSE, standard error estimate for the log2 fold change; stat, the value of the test statistic for the gene or transcript; pvalue, p-value of the test for the gene or transcript; padj; adjusted p-value for multiple testing for the gene or transcript.

Supplementary Table S6. Differentially expressed miRNAs on D9 after TBI (TBI+ *vs.* sham animals).

miRNA	baseMean	log2FC	lfcSE	stat	pvalue	padj
**Upregulated**						
rno-let-7c-2-3p	52.41	1.34	0.40	3.33	8.56E-04	4.10E-02
rno-let-7a-1-3p	52.03	1.40	0.41	3.45	5.70E-04	3.18E-02
**Downregulated**						
rno-miR-7a-5p	17.61	-5.96	1.27	-4.68	2.85E-06	9.55E-04
rno-miR-140-3p	5872.00	-1.20	0.28	-4.30	1.74E-05	2.92E-03
rno-miR-674-5p	9.79	-2.22	0.60	-3.72	2.02E-04	2.25E-02
rno-miR-149-5p	155.94	-1.21	0.34	-3.59	3.30E-04	2.76E-02
rno-miR-7b	7.77	-6.46	1.86	-3.48	5.03E-04	3.18E-02
rno-miR-326-3p	104.06	-0.97	0.29	-3.29	1.00E-03	4.20E-02
rno-miR-455-5p	92.70	-1.57	0.48	-3.25	1.16E-03	4.31E-02
rno-miR-351-5p	1098.20	-1.34	0.42	-3.22	1.30E-03	4.34E-02
rno-miR-25-5p	19.30	-1.86	0.59	-3.17	1.52E-03	4.64E-02

Abbreviations: baseMean, the average of the normalized count values; log2FC, log2 fold change; lfcSE, standard error estimate for the log2 fold change; stat, the value of the test statistic for the gene or transcript; pvalue, p-value of the test for the gene or transcript; padj; adjusted p-value for multiple testing for the gene or transcript.

Supplementary Table S7. Receiver operating characteristic (ROC) and cut-point analysis of circulating rno-miR-434-3p, rno-miR-9a-3p, rno-miR-136-3p, rno-miR-323-3p, rno-miR-124-3p, rno-miR-212-3p and rno-miR-132-3p between the animal groups (naïve, sham, TBI). ***Statistical significances***: *, p < 0.05; **, p < 0.01; ***, p < 0.001; ns, not significant.

**rno-miR-434-3p**				
Comparisons	ROC AUC	Cut-off (normalized expression)	Sensitivity (%)	Specificity (%)
naïve *vs*. sham	0.96***	0.22	82	100
naïve vs. TBI	1.00***	0.31	100	100
sham vs. TBI	0.98***	0.69	88	100
**rno-miR-9a-3p**				
Comparisons	ROC AUC	Cut-off (normalized expression)	Sensitivity (%)	Specificity (%)
naïve vs. sham	0.76*	0.04	53	100
naïve vs. TBI	0.99***	0.18	99	100
sham vs. TBI	0.97***	0.18	99	94
**rno-miR-136-3p**				
Comparisons	ROC AUC	Cut-off (normalized expression)	Sensitivity (%)	Specificity (%)
naïve vs. sham	0.88**	0.03	88	75
naïve vs. TBI	1.00***	0.05	99	100
sham vs. TBI	0.96***	0.07	93	88
**rno-miR-323-3p**				
Comparisons	ROC AUC	Cut-off (normalized expression)	Sensitivity (%)	Specificity (%)
naïve vs. sham	0.93***	0.04	76	100
naïve vs. TBI	1.00***	0.09	100	100
sham vs. TBI	0.99***	0.12	98	94
**rno-miR-124-3p**				
Comparisons	ROC AUC	Cut-off (normalized expression)	Sensitivity (%)	Specificity (%)
naïve vs. sham	0.49 (ns)	0.04	94	25
naïve vs. TBI	1.00***	0.20	100	100
sham vs. TBI	1.00***	0.26	99	100
**rno-miR-212-3p**				
Comparisons	ROC AUC	Cut-off (normalized expression)	Sensitivity (%)	Specificity (%)
naïve vs. sham	0.81*	0.33	59	100
naïve vs. TBI	0.95***	0.33	82	100
sham vs. TBI	0.76***	0.39	69	71
**rno-miR-132-3p**				
Comparisons	ROC AUC	Cut-off (normalized expression)	Sensitivity (%)	Specificity (%)
naïve vs. sham	0.79*	0.39	82	88
naïve vs. TBI	0.98***	0.60	89	100
sham vs. TBI	0.94***	0.65	87	94

Abbreviations: AUC, area under the curve; TBI, traumatic brain injury.

Supplementary Table S8. Receiver operating characteristic (ROC) and cut-point analysis of circulating rno-miR-434-3p, rno-miR-9a-3p, rno-miR-136-3p, rno-miR-323-3p, rno-miR-124-3p, rno-miR-212-3p and rno-miR-132-3p between TBI+ and TBI- groups and epilepsy severity groups. ***Statistical significances***: ns, not significant.

**rno-miR-434-3p**				
Comparisons	ROC AUC	Cut-off (normalized expression)	Sensitivity (%)	Specificity (%)
TBI+ *vs*. TBI-	0.55 (ns)	2.38	29	91
TBI+ <3 *vs*. TBI+ ≥3	0.58 (ns)	0.91	47	100
TBI+noC *vs*. TBI+C	0.51 (ns)	0.91	50	77
**rno-miR-9a-3p**				
Comparisons	ROC AUC	Cut-off (normalized expression)	Sensitivity (%)	Specificity (%)
TBI+ *vs*. TBI-	0.52 (ns)	1.38	33	87
TBI+ <3 *vs*. TBI+ ≥3	0.54 (ns)	1.94	20	100
TBI+noC *vs*. TBI+C	0.58 (ns)	0.31	100	23
**rno-miR-136-3p**				
Comparisons	ROC AUC	Cut-off (normalized expression)	Sensitivity (%)	Specificity (%)
TBI+ *vs*. TBI-	0.58 (ns)	0.22	43	77
TBI+ <3 *vs*. TBI+ ≥3	0.62 (ns)	0.12	40	100
TBI+noC *vs*. TBI+C	0.51 (ns)	0.22	75	46
**rno-miR-323-3p**				
Comparisons	ROC AUC	Cut-off (normalized expression)	Sensitivity (%)	Specificity (%)
TBI+ *vs*. TBI-	0.53 (ns)	0.77	33	88
TBI+ <3 *vs*. TBI+ ≥3	0.68 (ns)	0.29	53	100
TBI+noC *vs*. TBI+C	0.56 (ns)	0.29	63	77
**rno-miR-124-3p**				
Comparisons	ROC AUC	Cut-off (normalized expression)	Sensitivity (%)	Specificity (%)
TBI+ *vs*. TBI-	0.55 (ns)	2.31	33	88
TBI+ <3 *vs*. TBI+ ≥3	0.51 (ns)	2.35	33	83
TBI+noC *vs*. TBI+C	0.55 (ns)	0.77	88	38
**rno-miR-212-3p**				
Comparisons	ROC AUC	Cut-off (normalized expression)	Sensitivity (%)	Specificity (%)
TBI+ *vs*. TBI-	0.53 (ns)	0.62	48	74
TBI+ <3 *vs*. TBI+ ≥3	0.71 (ns)	0.36	47	100
TBI+noC *vs*. TBI+C	0.62 (ns)	0.42	63	69
**rno-miR-132-3p**				
Comparisons	ROC AUC	Cut-off (normalized expression)	Sensitivity (%)	Specificity (%)
TBI+ *vs*. TBI-	0.54 (ns)	1.75	23	99
TBI+ <3 *vs*. TBI+ ≥3	0.63 (ns)	0.95	60	83
TBI+noC *vs*. TBI+C	0.52 (ns)	1.75	38	85

Abbreviations: AUC, area under the curve; TBI, traumatic brain injury; TBI+, TBI rats with epilepsy; TBI-, TBI rats without epilepsy; TBI+ <3, TBI+ rats with < 3 seizures per month; TBI+ ≥3; TBI+ rats with ≥3 seizures per month; TBI+noC; TBI+ rats without seizure clusters; TBI+C, TBI+ rats with seizure clusters (≥3 seizures within 24 h).
